# Comparative functional profiles of microbial communities on drifting microplastics and volcanic pumice

**DOI:** 10.1093/ismeco/ycag158

**Published:** 2026-06-09

**Authors:** Shiye Zhao, Ryan P Bos, Ryota Nakajima

**Affiliations:** Institute for Earth and Materials Sciences (EMS), Japan Agency for Marine-Earth Science and Technology (JAMSTEC), Yokosuka, Kanagawa 237-0061, Japan; Department of Organismic and Evolutionary Biology, Harvard University, Cambridge, MA 02138, United States; Institute for Earth and Materials Sciences (EMS), Japan Agency for Marine-Earth Science and Technology (JAMSTEC), Yokosuka, Kanagawa 237-0061, Japan

**Keywords:** plastisphere, microplastic, pumice, microbial community, metagenomics, marine plastic debris

## Abstract

Plastics have been shown in incubation experiments to select for distinct microbial communities from biogenic and inanimate controls, with successional shifts over time. However, few field studies have directly compared microbial communities on free-drifting plastic debris and non-plastic particles. Using shotgun metagenomics, we analyzed the microbial communities adhered to marine microplastics and co-drifting volcanic pumice as a time-tracked control to investigate differences in metabolic potential. Overall, the mature microbial communities on neuston-net collected microplastics and pumice exhibited broad functional and taxonomic similarity, providing suggestive evidence of function convergence. Interestingly, plastic hydrolysis genes, and putative hydrocarbon-degrading bacteria were scarce on both substrates, whereas β-glucan degradation genes were abundant, indicating potential utilization of biofilm-associated carbon sources. Nevertheless, pumice biofilms exhibited substrate-associated enrichment of genes linking to biofilm formation, quorum sensing, nitrogen and phosphonate metabolism, suggesting expanded genomic versatility. Considering the increasing input of anthropogenic and natural inanimate particles may act as environmental perturbations, potentially shaping microbial succession and metabolic potential on floating surfaces. Our findings provide insight into the genomic potential of particle-associated assemblages that stay afloat for months to years, and their metabolic responses to both natural and anthropogenic perturbations.

## Introduction

Microplastics (1 μm–5 mm) represent an unprecedented perturbation to the ocean [1]. Once introduced into the marine environment, microbes, key players in ocean productivity and biogeochemical cycling [2], readily colonize microplastics, forming biofilms, known as the “Plastisphere” [3], similar to those on other solid surface [4, 5], thus posing latent threats to marine ecosystems [6]. A conservative estimate suggests that plastic debris afloat in the global ocean provides ~2.5 × 10^10^ m^2^ of surface area for microbial colonization, carrying up to 3 × 10^21^ microbial cells, roughly equivalent to 10^4^ metric tons of carbon biomass [7].

Studies of the Plastisphere have examined taxonomic composition and succession, and differences across different substrate types and ambient environments, as summarized in Fig. S1 based on a review of 122 studies. Most of these studies involved *in-situ* experiments where plastics (both conventional and biodegradable plastic polymers) and non-plastic controls (e.g. inanimate materials like glass, stone, ceramic and steel; and biogenic materials like cellulose and wood, Fig. S1) were immersed beneath the sea surface and remained at a fixed location without drifting. Comparing microbiomes on plastics with those on control substrates has revealed the specificity of biofilm communities on plastic surfaces and between biofilm phases [8–12]. Unlike incubation studies, few field-based investigations have compared microbial communities on plastics and natural substrates such as Sargassum and cellulose [13, 14]. Moreover, previous research primarily depended on marker-gene amplicon sequencing (Fig. S1), leaving the functional traits that mediate microbial interactions with substrates and the environment elusive. Recent studies using shotgun metagenomics, metatranscriptomics, and metaproteomics have begun to reveal the functional complexity of Plastisphere communities [9, 12,14–17]. Understanding the fate/trajectory of these communities on plastic particles that can persist for hundreds to thousands of years in the ocean enables us to understand their potential broader ecosystem impacts.

*In-situ* incubation experiments of the plastisphere have principally assessed the influence of surface material on biofilm development, while largely overlooking buoyancy and drifting behaviors that expose plastic-attached microbes to variable environmental conditions. For instance, varying degrees of solar radiation exposure, could influence marine microorganisms both directly by impairing cellular components and indirectly through photo-transformation of substrate materials and surrounding organic matter [18]. Indeed, one study incubating plastics and control glass beads in ambient and dim light, observed higher abundances of autotrophs (e.g. Cyanobacteria) under ambient light [8]. Bravo et al. (2011) used light and electron microscopy to explore how surface properties, buoyancy, and floating behavior of abiotic substrates (plastics and volcanic pumice) influence microbial colonization through a 98-day incubation experiment. They concluded that floating behavior and buoyancy play dominant roles in shaping microbial communities, whereas surface chemistry has only minor effects [19]. Therefore, including control substrates with similar physical behavior in the water column (e.g. buoyancy) to plastics is critical for teasing out unique functional contributions from the Plastisphere [9, 20, 21].

Volcanic pumice, mainly composed of inorganic silicate glass and filled with air bubbles, is the only known type of stone that floats at the sea surface and can drift across oceans for months to years [22]. Like many buoyant plastic particles, pumice serves as a durable substrate that enables long-lasting distance transport of marine species. For example, pumices that travelled over 5000 km in 7–8 months carried over 80 sessile species [23]. Pumice is also initially sterile due to its volcanic origin, making it an ideal inorganic, sterile control substratum for studying the genomic traits of microbes colonizing plastic surface. Sessile species have been identified microscopically [19,23–25], but molecular studies of pumice-attached microbial communities are lacking, despite their potential to reveal microbial responses to natural disturbances like volcanic eruptions.

The eruption of the Fukutoku-Oka-no-Ba volcano (Japan) on August 13th, 2021 generated pumice rafts [26], offering a rare opportunity to compare microbial communities on natural, inert particles of known age with those of free-drifting plastic particles of unknown age. To that end, we leveraged whole-genome-shotgun sequencing followed by metagenomics to compare the functional and taxonomic composition of free-drifting plastic and pumice communities. We also reconstructed metagenome-assembled genomes (MAGs) to investigate substrate-associated differences in genome architecture. Parallel analyses of surrounding seawater metagenomes provided additional microbial ecological context.

## Materials and methods

**Figure 1:**
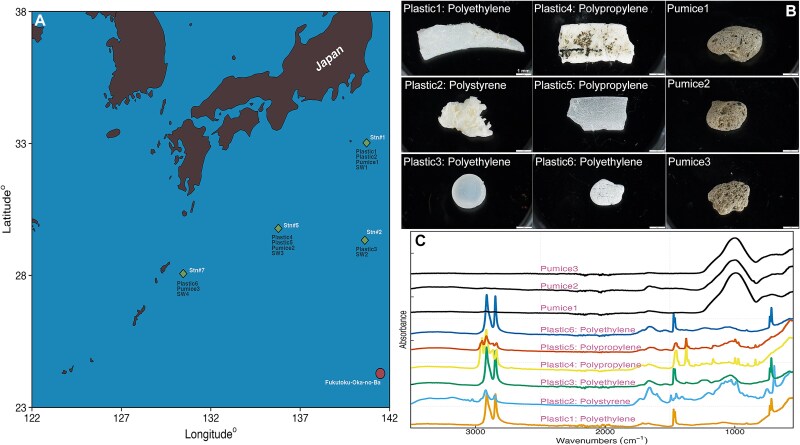
Information of particles analyzed in this study. A. Locations of sampling stations along the Japan coast. The neuston net was deployed at 12 stations. DNA samples were collected at four stations. The red dot represents the location of the Fukutoku-Oka-no-Ba volcano, which erupted on August 13^th^, 2021. B. Optical microscopy of microplastic and pumice particles extracted for DNA analysis. C. Fourier transform infrared spectroscopy spectra of particles used.

Sampling was conducted at four stations along the Japan coast (Fig. 1A) during the leg from Yokohama to Ishigaki (September 16 to 30, 2022) aboard the Norwegian tall ship *Statsraad Lehmkuhl* as part of the One Ocean Expedition 2021–2023 circumnavigation [27]. Floating particles were collected using a neuston net (mesh size, 333 μm; mouth opening size, 1.0 m width and 0.75 m height) equipped with a calibrated flow meter (Rigo, Japan). The neuston net was deployed from the ship’s starboard side using a davit and towed for ~20 min at a speed of 1–2 knots at the sea surface, where samples were collected in the cod-end bag of the net [28]. Once onboard, the cod-end bag was detached, and the content was washed off by immersing the bag into a 70% ethanol sterilized bucket with 0.22 μm filtered surface seawater. Pre-disinfected tweezers were used to pick up particles, which were gently rinsed with 0.22 μm filtered surface seawater to remove sloughed-off cells. Finally, the particles were placed into individual 2-ml cryovials with 1.5 ml of 1× DNA/RNA Shield (Zymo Research Corp., USA). At each station, 4 liter of surface seawater was filtered through 0.22 μm Luer-lok Sterivex™ filters (EMD Millipore, Italy) to collect free-living bacteria. Then, 1.5 ml of 1× DNA/RNA Shield was injected into the Sterivex filter. Particles in cryovials and Sterivex filters were kept chilled on blue ice after collection until being frozen (at −80°C) upon return to the lab.

### DNA extraction

ZymoBIOMICS DNA Kit was used to extract DNA. Briefly, after thawing at room temperature, each particle (microplastic or Pumice) in a cryotube with 1.5 ml DNA/RNA Shield was transferred into a 2 ml ZR BashingBead™ lysis tube containing beads using clean forceps. The remaining 1.5 ml DNA/RNA Shield from the cryotube was then pipetted into the same lysis tube. The tubes, each containing individual particle, were placed on a vortex microtube adapter that holds 1.5–2.0 ml tubes and were homogenized on the vortex mixer at maximum speed for 40 ~ 60 min to disrupt the cell walls. Further DNA purification was conducted following the manufacturer’s instruction. The DNA extraction from filters resulting from seawater filtration were also performed using the kit as described above. Finally, the isolated DNA was stored at -80°C for further analysis.

### Whole-genome-shotgun sequencing

Whole-genome-shotgun sequencing was carried out by the Bioengineering Lab. Co., Ltd. (Sagamihara, Japan) using DNBSEQ-G400 platform (MGI Tech, Shenzhen, China). A sequencing library was prepared using an MGIEasy FS DNA library prep set (MGI Tech Co., Ltd.) or Nextera XT DNA Library Prep Kit (Illumina) (Table S1). To assess potential bias, the effect of the different library preparation kits on the taxonomical and functional compositions was analyzed via PERMANOVA tests; no significant influence was detected (Table S2). Circularized DNA and DNA nanoballs were prepared using an MGIEasy circularization kit (MGI Tech) and a DNBSEQ-G400RS high-throughput sequencing kit (MGI Tech), respectively. Paired-end (2 × 200-bp) sequencing on a DNBSEQ-G400 sequencer (MGI Tech) produced 438 487 454 raw sequence reads (total of 106 997 000 000 bp).

### Bioinformatic approach

Paired-end read sets were trimmed and quality-filtered using bbduk.sh within the BBMap platform (v.39.06) [29] in two passes. The first pass used parameters “ref” MGI adapters “ktrim = r k = 23 mink = 11 hdist = 1 tbo tpe” to remove sequencing adapters. The second pass used parameters “k=27 hdist=1 qtrim=rl trimq=17 cardinality=t mingc=0.05 maxgc=0.95” to remove low-quality bases and sequences with abnormally high or low GC content. Additional low-quality bases and sequences were removed with Trimmomatic 0.38 (parameters: LEADING:10 TRAILING:10 MINLEN:100) [30].

Taxonomic classification was performed using Kraken 2 (v.2.0.8-beta) with the standard Kraken 2 database [31]. Bracken v.2.5.0 was used to re-estimate abundance for calculating the alpha and beta diversity of bacterial communities [32]. Genera that were common contaminants in the laboratory environment but not representative of marine bacteria, such as *Staphylococcus* (mean 0.065%) and *Bradyrhizobium* (mean 0.005%, Table S3), were removed. We acknowledge that post-hoc filtering is not an alternative to field or extraction blanks; consequently, results regarding the presence or absence of rare taxa and low-abundance functions are interpreted with caution.

Quality reads from each sample were individually assembled using metaSPAdes v.3.15.5 with default parameters and k-mer length 21, 33, 55, 77, 99, 127 on the Galaxy platform [33]. The open reading frames (ORFs) of contigs that were above 300 bp in length, were predicted using Prodigal (v.2.6.3) [34]. ORFs from all samples were clustered to generate a non-redundant gene set using MMseqs2 [35]. Functional annotations of the non-redundant gene set were conducted using Diamond to align ORFs against eggNOG v.5.05 with the DIAMOND search [36]. The emapper command was run with default parameters. The eggNOG-mapper output was parsed to extract genes with assigned KEGG Orthology (KO) numbers, gene names, and relevant functional descriptions. A KO reference table, including three KEGG levels, KO numbers, and gene names, was generated from the ko.json file downloaded from the KEGG website. Functional annotations were then matched with this KO table to produce a final dataset containing non-redundant genes, KO numbers, gene names, and KEGG levels. All filtering steps were performed using custom-made Python scripts. The DNA-based gene abundance in each sample was estimated by the number of mapped reads and normalized: reads per kilo base per million mapped reads (RPKM) = number of Reads / (geneLength/1000 × total Number of Reads/1000000). The reads were mapped to the non-redundant gene set with BWA with default parameters [37], and the RPKM value of each gene in each sample was calculated to compare the change in the gene abundances in different samples.

Preprocessed reads were mapped to metagenomic assemblies with Bowtie 2 (v.2.3.2) with default options [38]. MAGs were binned using Metabat2 v.2.12.1 (jgi_summarize_bam_contig_depths with options –minContigLength 1500) [39]. The completeness and contamination of each bin was assessed with CheckM v.1.2.2 using the lineage_wf workflow [40]. MAGs with an estimated genome completeness >70% and contamination <3% were included in downstream analyses. Taxonomic classification of all MAGs was determined using GTDB-TK (v.2.0.3.2) [41]. For the functional annotation of selected MAGs, genes are called and annotated using Prokka v.1.13 using default options [42] and the estimated domain classification from CheckM as the argument in the –kingdom option. Additionally, ORFs were annotated with eggNOG v.5.05, as described above. Carbohydrate-Active enzymes were annotated by running dbCAN (v.3.0.7) with HMMER 3.4 (default E value) [43].

### Statistical analysis

Statistical analysis and visualization of the data in this study were performed using R v.4.1.1 [44]. Alpha and beta diversity analyses were conducted using the “vegan” package in R. The permutational multivariate analysis of variance (PERMANOVA) using the Adonis test (999 permutations) in R package “vegan” was conducted to reveal the differences between samples. For one variant, the Kruskal–Wallis test was used for multiple group comparisons, followed by pairwise comparisons using the nonparametric Mann–Whitney U test. Significance level at *P* < .05 was employed. Given the modest sample size, we conducted post-hoc power checks based on observed effect sizes to evaluate our ability to detect differences between substrates. Specifically, we assessed multivariate power for taxonomical and functional profiles (PERMANOVA R [2], 999 simulations) via resampling-based simulations and estimated univariate power for selected gene abundances using Cohen’s d and classical power calculations. The “ggplot2” package was used to produce all graphics in this study.

## Results and discussion

In this study, free-drifting pumice particles of known age (~13 months) and marine microplastics (>300 μm) of unknown age were both presumed to harbor mature biofilms. For the free-drifting microplastics, the former notion was substantiated by the extensive discoloration, mechanical weathering showed in micrographs (Fig. 1B), and substantial biofilms environmental DNA extracted from each microplastic piece, indicating the persistence of older biofilms. Additionally, the detection of carbonyl groups, chemical signs of oxidative aging, in all measured spectra of free-drifting microplastics further supports the presence of late-stage biofilms (Fig. S2). While we recognize the constraints imposed by a limited sample size, statistical power analyses confirm that the functional and taxonomic similarities between plastic- and pumice-associated communities provide suggestive evidence of functional convergence. This supports new insights into microbial responses to natural and anthropogenic perturbation events.

### Microbial diversity

**Figure 2:**
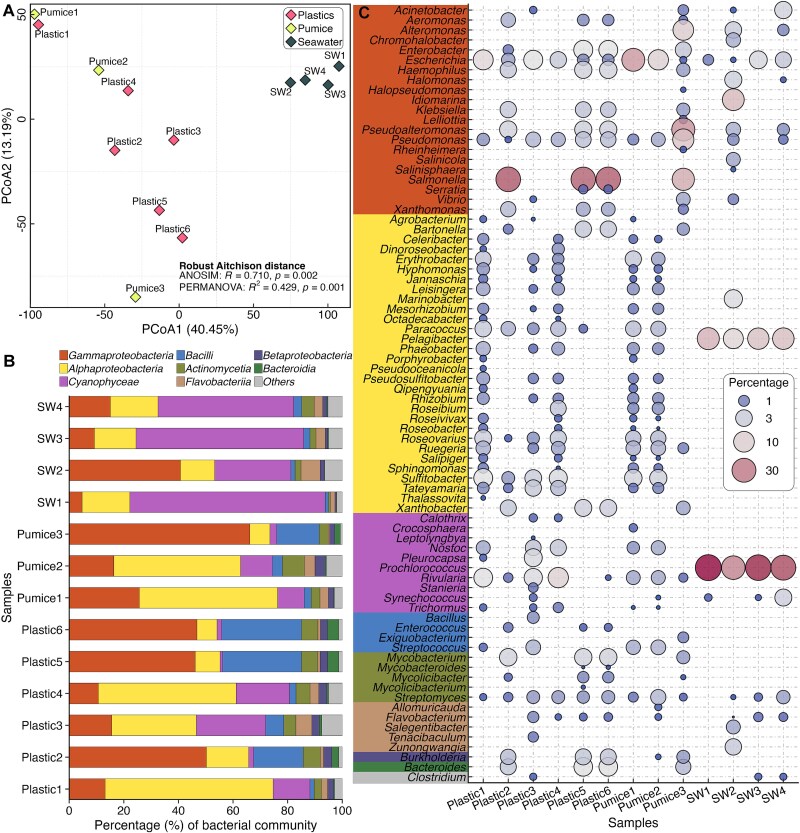
Taxonomic profiling of the bacterial community composition colonized on floating plastics and pumices, and in the surrounding seawater. A. PCoA of communities at species level from plastics, pumices and seawater based on robust Aitchison’s distance. Results of ANOSIM and PERMANOVA tests are shown in the box. “SW” represents seawater samples. Statistical powers for the ANOSIM and PERMANOVA tests are 0.721 and 0.772, respectively. B. Bar chart showing the relative abundance of bacterial groups at the class-level. Classes with abundances of <1% in one sample are shown in “others.” C. Bacterial genera with abundance >0.5% among all metagenomes.

A total of 13 metagenomes were assembled from samples collected across four stations (Fig. 1, Table S1), including six from microplastics, three from pumices and four from seawater. Each metagenome was composed predominantly of bacterial reads, ranging from 96% to 99% out of total reads (Table S3). In accordance with previous field and incubation studies [3, 16, 45], the compositions of bacterial community on floating plastics and pumices in our study were distinct from those in the surrounding seawater (Fig. 2). Principal Component Analysis (PCoA) showed that surface attached and free-living samples were distinguishable (PERMANOVA, R^2^ = 0.429, *P* = .001; ANOSIM, R = 0.710, *P* = .002, Figs. 2A and S3). The seawater samples were separated from all substrates on the first axis (representing 40.45% variation) and the two floating substrates were separated on the second axis (13.19% variation, Fig. 2A).

The metabolic and phylogenetic distinctiveness of free-living and surface-associated microbes has been well studied, leading to the conclusion that specific bacteria colonizing surfaces may be influenced by the physical and chemical compositions of their environment (e.g. nutrient and energy availability) as well as their microhabitat-specific adaptive traits (e.g. surface-associated competitive and synergistic ecological interactions) [46, 47]. The dominance by the class of Cyanophyceae discriminate the seawater metagenomic samples over those associated to plastics and pumices (Fig. 2B). The genus *Prochlorococcus* expectedly dominated free-living cyanobacteria [48], with relative abundance from 30% to 72%. *Pelagibacter* spp. in seawater, accounting for 8% to 16% (Fig. 2C), also contribute to the distinction between particle-associated and free-living communities. This aligns well with previous observations [3, 49]. In contrast, the cyanobacterial genera *Rivularia* and *Nostoc*, either accounted for a relative abundance of less than 10% on plastic and pumice surfaces (Fig. 2C). These genera were consistently reported in marine Plastisphere communities [3, 50]. Furthermore, microbial assemblages in the bulk seawater represent only a snapshot of the community present at the time of sample collection. In contrast, biofilm communities on the floating substrates are a cumulative and successional community developed over unknown durations [51].

No apparent taxonomic difference between plastic and pumice substrates is observable (Figs. 2A, S3, and  S4), and this observation is in accordance with data from field-collected and incubation samples comparing microorganisms residing on plastics and other substrates such as wood and glass [10, 13,52–54]. Within a given environment, the microbial community on plastics and other substrates converge over time as the biofilms progress toward maturity [51, 55]. One study incubated four types of plastics (polyvinylchloride, polypropylene, low-density and high-density polyethylene) and glass in the coastal Northern Adriatic to determine the substrate specificity of microbial communities. The authors showed that differences in bacterial communities between different polymer types and between plastic and glass were more apparent at early stage (1 week of incubation) than later stage (after 1–2 months of incubation) [8]. No distinctions of bacterial communities between substrates at later stage biofilms suggest that the influence of the physiochemical characteristics of the substratum surface (such as hydrophobicity, surface texture, chemical nature and plastic leachate) on the adhesion of microbes is only apparent at the initial stages and disappears with maturation of the biofilm [7, 8]. Previous incubation experiments suggest that a mature biofilm communities on plastics and other substrates can be established just over 1 week [51, 56]. In our samples, the pumice stones, originating from the Fukutoku-Oka-no-Ba volcanic eruption on August 13, 2021, had been adrift in the ocean for ~13 months by the time of our collection between September 16 and 30, 2022. This duration of drifting pumices suggests that their attached biofilms had likely progressed past the initial stages of development. The similar taxonomic composition on plastic and pumice particles (Fig. 2A) may be attributed, at least in part, to the surface-associated biofilms being in mature or late developmental stages. Apparent cracks and discoloration on microplastic particles, along with the presence of carbonyl species in the range of 1850–1650 cm^−1^, suggest that the plastic particles were highly aged (Figs. 1B and S2), possibly having remained in the marine environment for an extended period and thereby harboring more advanced biofilms. Although these particles harbor mature biofilms, subtle differences in taxonomic composition may arise from the unique life history characteristics and trajectories of these surfaces. This variation may explain the partial separation of bacterial communities on plastics and pumices along the second axis of the PCoA (Fig. 2A). For example, different drifting behaviors, such as rolling or floating in a consistent orientation, expose various particle surface to different levels of solar irradiation, ultimately influencing biofilm formation and removal on surfaces [57]. Through scanning electron microscope observations, one study demonstrated that drifting and floating behaviors have substantial impacts on the community succession on abiotic [19]. As shown in Fig. 1B, the morphological properties of plastic and pumice particles, such as cracks, surface roughness, shape factors, vary substantially, which influences their drifting behavior [58]. In addition, the proposed erase-and-reset mechanism [59], involving mechanical disruption (e.g. wave-driven particle abrasion) and predation on biofilms (e.g. by grazers), could also account for community nuances.

Several enteric taxa, including *Escherichia, Salmonella*, and *Klebsiella*, were detected at elevated relative abundances in a subset of particle-associated communities (Fig. 2C). Plastic surfaces are increasingly recognized as reservoirs and vectors for pathogenic and fecal indicator bacteria in aquatic environments influenced by wastewater discharge [60, 61]. Members of the Enterobacteriaceae family have previously been reported as colonizers of plastic surfaces in freshwater and coastal systems [62, 63]. Although potential reagent or laboratory contamination cannot be completely excluded, several observations support the environmental plausibility of these taxa in our dataset. First, their distribution was unevenly across substrates and stations rather than consistently detected at low abundance across all samples, which is less characteristic of typical reagent contamination. In addition, all particle-associated DNA extractions were processed within a single batch, reducing the likelihood of batch-specific contamination effects.

### Functional potential

**Figure 3:**
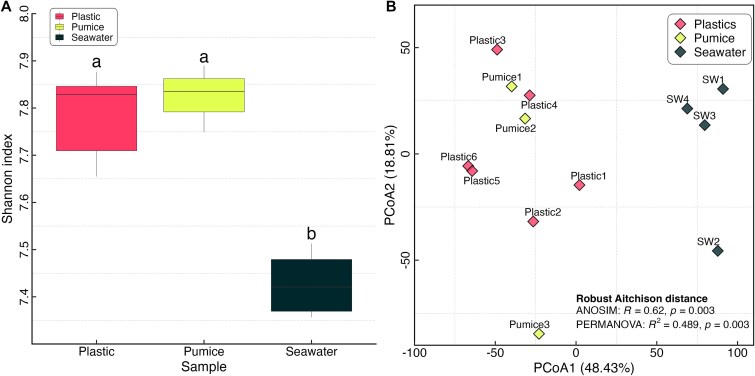
Comparison of potential functioning in different metagenome samples. A. The Shannon index of samples determined based on the KOs abundances (RPKM values). Significance of results was evaluated using Kruskal-Wallis test (*P* = .02, statistic power = 0.71) and Mann–Whitney U tests and labeled using different letters. Statistical powers for the pumice vs. seawater comparison, and the plastic vs. seawater comparison are 1.0 and 0.98, respectively. The center line represents the median; box limits indicate the first and third quartiles; and whiskers show 1.5 times the interquartile range. B. KOs abundances-based PCoA plots of the Robust Aitchison’s distance. Results of ANOSIM and PERMANOVA tests are shown in the box. The statistical power to detect the observed differences was calculated to be 1.0 for both the ANOSIM and PERMANOVA tests.

A total of 17 585 unique KO identifiers (IDs) were identified across microplastics, pumices, and seawater metagenomes. KEGG level-3 pathway analysis revealed diverse metabolic potential, including transporters, two-component system, DNA repair and recombination proteins, and quorum sensing (Fig. S5). Functional diversity, estimated by the Shannon index of KO abundance, was significantly higher in particle-attached than in seawater communities (Kruskal–Wallis test, *P* = .02, Fig. 3A). This suggests that biofilm-associated assemblages harbor more functionally diverse and redundant gene repertoires, consistent with their complex microenvironments [64]. Principal coordinates analysis based on Robust Aitchison’s distance showed that the functional structures of particle-associated communities was significantly distinct from that of seawater (PERMANOVA, R^2^ = 0.489, *P* = .003; ANOSIM, R = 0.62, *P* = .003, Fig. 3B). However, plastics and pumices were not clearly separated, indicating overall functional similarity between substrates. This pattern agrees with previous metagenomic studies that reported comparable particle-attached metabolic profiles distinct from free-living communities [16, 47, 65]. These findings highlight that bacterial community composition and functional similarity were coupled, with functional structure largely shaped by community lifestyle [65].

Higher normalized counts of phycobilisome antenna protein-encoding genes were observed in biofilm communities on plastics and pumices compared to seawater (Kruskal-Wallis and Mann–Whitney U tests, *P* < .05, Fig. S6). Phycobilisome serve as the dominant light-harvesting antenna in cyanobacteria and some algae [66], consistent with the presence of cyanobacteria in our samples (Figs. 2AB and S4). Elevated antenna protein abundance, which were also reported in previous studies [9, 16], may reflect adaption of photosynthetic microorganisms to fluctuating or reduced light conditions due to the hydrodynamic behaviors of drifting particles, particle structures or biofilm structures [67, 68]. This may be an evolutionary strategy to maximize light capture, supporting other heterotrophic bacterial metabolism within biofilm communities. In biofilms, the organic matter utilized by bacteria changed from photoautotrophic/autochthonous carbon at high light illumination to more complex allochthonous carbon at the dim light condition, which could alter the community structure and function of biofilms [69–71]. These finding evidences that floating surface-attaching microbes are sensitive to the solar irradiation and highlights the need to use materials with similar buoyancy and transport dynamics as floating plastic particles to accurately assess the ecological role of the Plastisphere.

Comparison of summed KO abundances at KEGG level 3 revealed that all significantly enriched pathways occurred in pumice-associated metagenomes. These pathways include lipid metabolism, xenobiotics biodegradation and metabolism, metabolism of terpenoids and polyketides, glycan biosynthesis and metabolism (Fig. S7). Enrichment of “alpha-linolenic acid metabolism” in lipid metabolism indicates enhanced potential for lipid remodeling and complex organic compound degradation in pumice biofilms (Fig. S7A). Pathways such as “Bisphenol degradation” and “Steroid degradation” suggest a higher potential of pumice-associated microbes to degrade complex organic compounds (Fig. S7B). Increased representation of secondary metabolites biosynthesis (e.g. “Biosynthesis of siderophore group nonribosomal peptides”, “Sesquiterpenoid and triterpenoid biosynthesis” and “Biosynthesis of type II polyketide products”, Fig. S7C) further suggests that biofilms on pumice are potentially producing more secondary metabolites. More abundant sequences of pathways related to “Glycosaminoglycan degradation” and “O-Antigen repeat unit biosynthesis” suggests that pumice microbes might have more complex cell surface structures or extracellular matrices (Fig. S7D). These distinct gene levels might be related to the distinct physical properties of plastics from pumices. Compared to plastic particles (Figs. 1B and S8), pumices, having highly porous structures and larger specific surface area, represent a more complex microhabitat that may support a more diverse and competitive mature biofilm community. In addition, compared to the complex chemical characteristics of plastics (e.g. hydrophobicity, compounds leached from plastic additives and oligopolymers), naturally sterile pumices are chemically inert. This also suggests chemical properties of substrate, which are more relevant for initial biofilm [7, 12], have minor influence on later biofilms [45, 56, 72].

**Figure 4:**
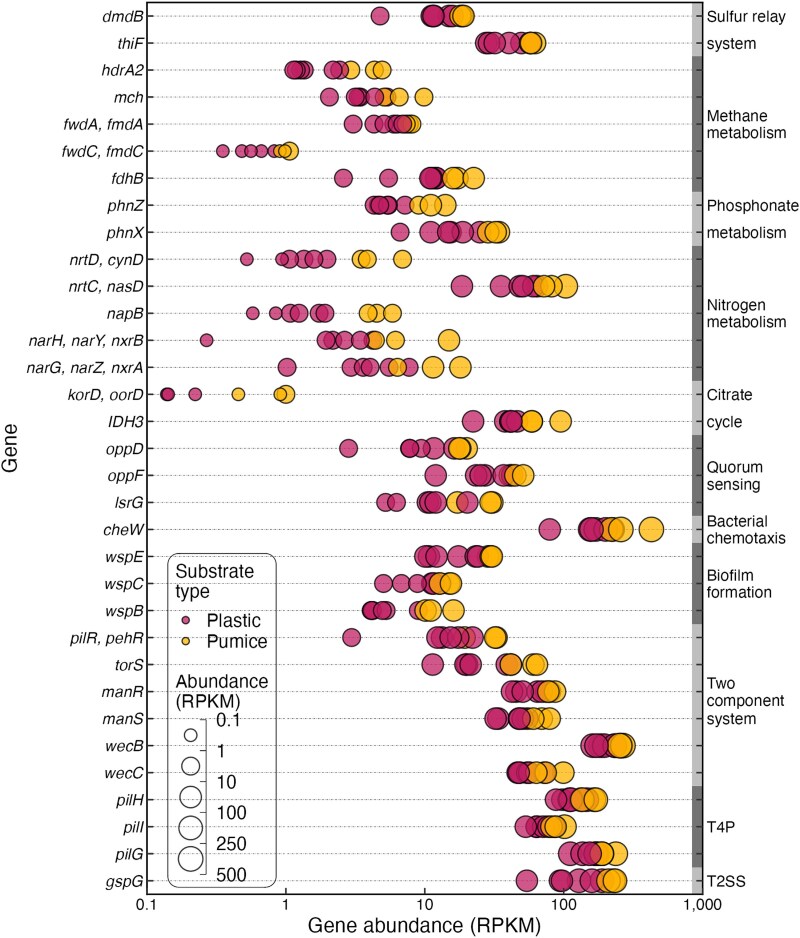
Abundances of selected critical genes significantly more abundant in pumice metagenomes compared to plastic metagenomes. Statistical significance (*P* < .05) was assessed using Mann–Whitney U tests. Statistical power for each gene ranges from 0.6 to 1 (Supporting dataset 1).

Consistent with these findings, at the gene level, the higher relative abundance of chemotaxis, two-component system, biofilm formation, type II secretion system, type IV pilus and quorum sensing-related core genes (Fig. 4), which regulate bacterial transitions between planktonic and biofilm states, twitching motility, DNA uptake, and surface attachment, as well as interaction with the environment [73, 74], suggests that pumice surfaces favor biofilm community development. In pumice-associated microbes, the enrichment of *oppD, oppF* genes, which provide energy for peptide translocation, suggests a higher genomic capacity for the Opp transporters. These transporters play a nutritional role by supplying bacteria with oligopeptides that serve as amino acid sources [75]. This indicates that pumice-attached microbial communities may have enhanced peptide uptake and nutrient acquisition capacity. Interestingly, higher gene abundances of nitrogen and phosphonate metabolism genes in pumice biofilms suggests that pumice-associated microbes exhibit greater potential for elemental cycling than those on plastics (Fig. 4). For example, the most common phosphohydrolase genes, *phnX* and *phnZ*, which are critical for liberating inorganic phosphate for cellular use by cleaving the carbon-phosphorus bond in phosphonates, showed higher abundances (RPKM) in pumice communities, indicating higher genomic potential for phosphonate cycling [76]. Following the volcanic eruption, large quantities of pumice entered the local marine ecosystem, posing negative impacts [25]. How this sudden influx of natural particles influences biogeochemical elemental cycling warrants further investigation. The estimated genome size for pumice-associated MAGs (6.1 ± 2.5 million base pairs, Mbp) was larger than that of plastic-associated MAGs (4.2 ± 1.8 Mbp; Mann–Whitney U tests, W = 62, *P* = .03; Fig. S9), suggesting broader gene repertoires and greater metabolic versatility [77]. Although potential methodological biases (e.g. assembly, binning, quality check of MAGs) should be considered, these findings collectively indicate that pumice-associated microbes exhibit expanded metabolic, regulatory capacities, reflecting adaptation to the drifting, heterogeneous and porous nature microhabitats. It is important to note that while our metagenomic data indicate the potential for various metabolic activities, further studies using RNA or protein analyses are needed to confirm their actual *in situ* metabolic functions.

### Putative plastic biodegradation

**Figure 5:**
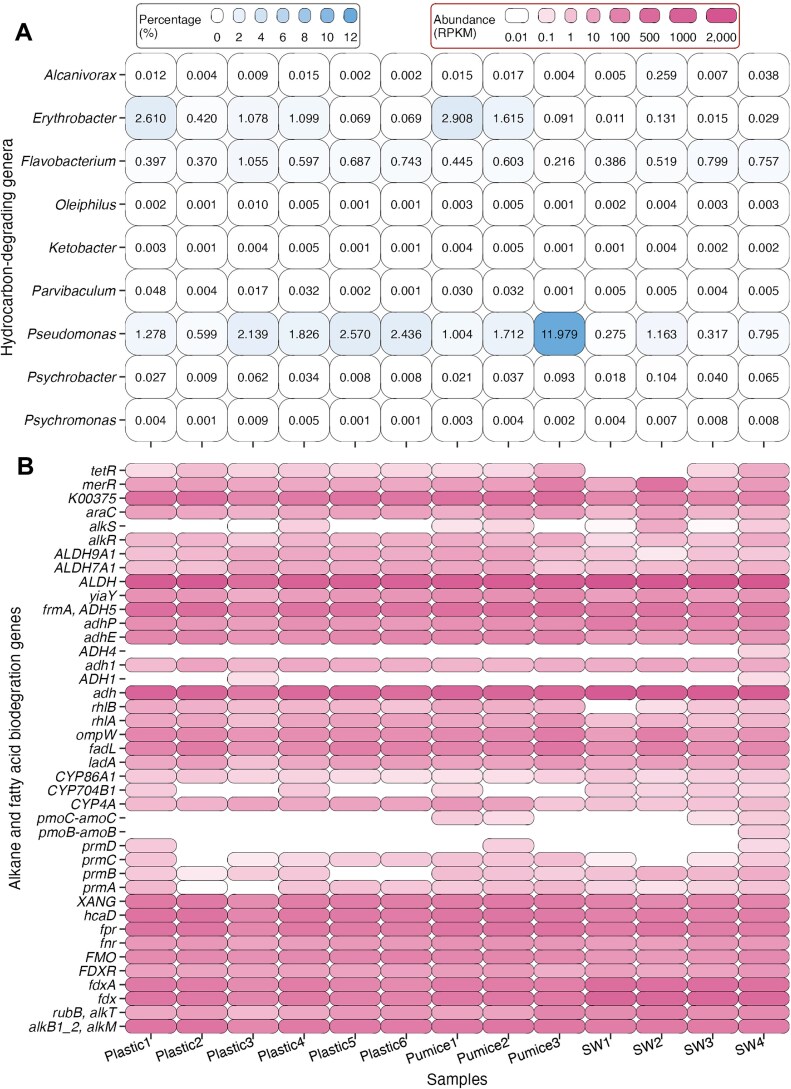
Potential plastic biodegradation by microbiomes across different samples. A. Relative abundances of obligate hydrocarbon-degrading bacterial genera in each metagenome sample. X-axis labels match those in the lower plot. Selected genera were drawn from the literature [56, 76]. B. Relative gene abundances (RPKM) of selected genes involved in alkane and or fatty acid degradation, as described in the study [78]. Empty areas are indicative of gene absence.

Given that common plastics are derived from hydrocarbons, one might expect adhered microbial communities to be enriched with obligate hydrocarbon-degrading bacteria (OHCB). In marine environments, OHCB are typically present in low abundance but can rapidly proliferate following a massive influx of hydrocarbon, such as during an oil spill [78]. In our study, several hydrocarbon-degrading bacterial groups, previously reported in the literature [56, 79], were identified on both substrates (plastic and pumice) and in seawater, with relative abundances ranging from 0.001% to 12% (Fig. 5A). However, no significant enrichment of these genera was found on plastic substrates (Fig. S10, Kruskal-Wallis test, *P* > .05, Table S4). Apart from *Pseudomonas*, which commonly dominates during both early and late colonization [80], OHCB taxa remained rare within Plastisphere (mostly <1%, Figs. 5A and S10), aligning with previous observations [13, 20, 56]. All plastic substrates examined in this study, including polyethylene (PE: Plastic1, 3 and 6), polypropylene (PP: Plastic4 and 5), and polystyrene (PS: Plastic2), consist of aliphatic C-C backbones that lack weak chemical bonds susceptible to enzymatic attack, making them largely refractory to microbial degradation [81]. Supporting this, we found no apparent increases in the relative abundances (RPKM) of the nine selected gene groups associated with alkane and fatty acid degradation on plastics compared to pumices and seawater (Figs. 5B and S11). Additionally, two MAGs were identified as belonging to the obligate hydrocarbon-degrading genus *Erythrobacter* (Figs. 5A and S12). The relative abundances of alkane and fatty acid degradation genes across all MAGs within their respective substrates (plastics/pumices) did not differ significantly (Kruskal-Wallis test, *P* = .72, Fig. S13). In a two-year experiment using low-density PE as the sole carbon source, these nine gene clusters were shown to be involved in its degradation [82]. While we cannot assess the initial biofilms that formed on our free-drifting plastics, our data of mature biofilms suggest that plastic moieties likely do not serve as a major carbon or energy source for the established microbial community; a finding that is consistent with previous data [56, 83].

**Figure 6:**
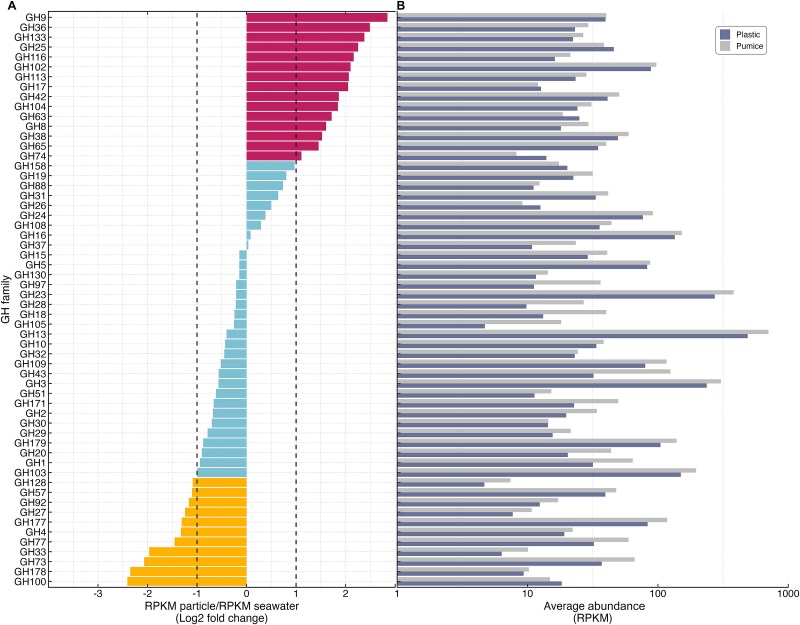
Gene abundances of GH families in particle-attached and free-living metagenomes. A. Changes of GH expression are indicated as a ration of RPKM in particle-attached metagenomes to free-living metagenomes. Only GH families with an average gene abundance greater than 10 RPKM in either particle or seawater samples were plotted. values are represented as log2-fold change. B. Mean gene abundances of GH families in RPKM in plastic- and pumice-associated metagenomes, respectively.

The early-stage enrichment of hydrocarbonoclastic taxa on plastics at early biofilm stages has been linked to their consumption of chain-scission compounds from weathered plastics, like plastic-derived dissolved organic carbon [84–86]. As biofilms mature, typically requiring at least one week as suggested by incubation experiments [51, 56], non-degrading microorganisms replace OCHB, consuming labile organic matter generated by biofilm-associated microbes rather than directly utilizing recalcitrant plastics [56, 82]. This shift in metabolic niches is further supported by the observed higher ratios of TonB-dependent transporters (TBDTs) to ATP-binding cassette (ABC) transporters within biofilm communities compared to seawater (Fig. S14, Kruskal-Wallis test, *P* = .03). ABC transporters and TBDTs are the two primary transporter types facilitating active exchange of organic matter between the environment and heterotrophic prokaryotic cells. TBDTs, often specific for mono- and polysaccharide uptake, serve as proxies for bacterial polysaccharide consumption [87]. Polysaccharide, major components of the biofilm matrix, can be degraded by various exocellular enzymes [4]. Along with other carbohydrates released by cells within the biofilm, these compounds can be utilized by neighboring microbes, facilitating metabolic cross-feeding [88]. This suggests that in mature Plastisphere communities, bacteria may preferentially rely on biofilm-associated organic matter rather than on plastic substrates [16]. The CAZymes results support the finding above. The PCoA analysis shows that the glycoside hydrolases (GH) families in particle-associated metagenomes are distinct from those in the seawater (Fig. S15), a proxy for polysaccharide degradation [89]. In particle-associated bacteria, the degradation of cellulose (β-1,4-glucans) as a carbon, was the most prominent by an increased normalized gene counts of GH families GH9, GH8, GH74 (magenta bars in Fig. 6A). The more abundant genes of GH17 are indicative of the degradation of β-1,3-glucans, possibly suggesting roles in fungal-derived polysaccharide. GH25, GH102, GH104 (peptidoglycan hydrolases) were higher expressed in particle-associated metagenomes, which contribute to the recycling of bacterial cell compounds as well as cell lysis phenomena in bacterial population [90], indicating possible bacterial predation or competition within the biofilm. It has been suggested that deep-sea microbes utilize peptidoglycans as a primary nutrient source [91]. Notably, the genes of these enriched GH families are all substantially expressed on both plastic and pumice substrates (Fig. 6B). By contrast, GH families enriched in seawater metagenomes are toward small, soluble carbohydrates. For example, genes encoding GH100, GH77, GH4, GH57 were enriched in seawater communities, which degrade soluble oligosaccharides α-glucans like glycogen [89].

### Potential biogeochemical implications of massive particle introductions

Our genomic analysis comparing mature biofilm communities on floating particles with free-living microbial communities suggests that the massive influx of particles into the ocean may perturb marine ecology and biogeochemical cycling. Specifically, elevated gene abundances related to phycobilisome antenna proteins in particle-associated communities indicate a genomic capacity for potentially enhanced photosynthetic adaption by cyanobacteria on surfaces (Fig. S6). This may theoretically influence CO₂ sequestration and influence other elemental cycles, such as nitrogen. Natural and synthetic particles also offer surfaces that could potentially alleviate nutrient limitations by concentrating trace nutrients like nitrogen, iron and phosphorus [5]. This may provide nutritional advantages for colonizing microbes, leading to a speculative potential for localized nutrient drawdown in seawater [7].

Our data show that pumice-attached communities harbor higher genomic potential (Figs. 4 and S7), and larger estimated MAGs genome sizes (6.1 ± 2.5 Mbp) compared to those on microplastics (4.2 ± 1.8 Mbp) (Fig. S9), potentially suggesting a greater nutrient demand with these samples [92]. Furthermore, the detection of *Mastigocoleus* MAGs on pumice (Fig. S12), a genus known to bio-erode marine carbonates [93], hints at a possible link between pumice-associated microbes to marine calcium cycle. Mineralogical analyses from a prior study indicates that pumices in our study (Fig. S8) are primarily composed of plagioclase and clinopyroxene phenocrysts, with CaO making up ~7–21% of their compositions [26]. Upon exposure, CaO can react with CO_2_ to form CaCO_3_, providing a substrate for endolithic *Mastigocoleus* [93].

## Conclusions

This study, for the first time, used field-collected, inorganic pumice particles as time-tracked controls to assess potential variations in microbial metagenomes associated with buoyant, synthetic microplastics. Our comparative genomic analyses revealed that overall microbial community composition and core functional profiles were broadly similar between microplastic (>300 μm) and pumice biofilms (Figs. 2 and 3). These findings support the view that the chemical properties of the underlying substrate may play an increasingly limited role in shaping the microbial composition and function as biofilm mature. This is further evidenced by the scarcity of plastic biodegradation genes and the absence of putative hydrocarbon-degrading bacteria and the elevated polysaccharide degradation genes on both substrates (Figs. 5, 6, S10, and  S11), indicating that plastic-derived carbon is unlikely to serve as a major energy source for mature biofilm communities. At the same time, pumice biofilms, however, showed substrate-associated enrichment in genes suggestive of higher metabolic potential (Fig. 4). This is probably attributed to pumice’s distinct physical traits including such as its porosity and high specific surface area, which likely support more complex and heterogeneous microbial habitats. Large-scale introductions of inanimate particles into marine ecosystem act as environmental perturbations, potentially driving shifts in microbial succession and metabolic potential on these foreign surfaces, with consequent impacts on biogeochemical processes. Additionally, our data highlights that surface-attached microbes are sensitive to solar irradiation (Fig. S6), emphasizing the importance of using substrates with buoyancy and transport characteristics similar to plastics when studying the Plastisphere.

Statistical power analyses support the robustness of the observed results between plastic- and pumice-associated communities; however, the small sample size may limit the generality of these results, underscoring the need for broader spatial and temporal investigations. While our study offers a snapshot of mature particle-associated biofilms with similar overall profiles but different life histories based on metagenomic data, future work should apply multi-omic approaches (e.g. metatranscriptomics, and metaproteomics) to investigate how the metabolisms of both microbes and eukaryotes on the surface of different particles and their interactions contribute to the effects of particle amendment on the ocean system.

## Supplementary Material

Supplementary_materials_ycag158

## Data Availability

Sequencing data generated and analyzed in the present study have been deposited at the NCBI Sequence Read Archive under BioProject ID: PRJNA1293120.
