# Supplementary material for: Comparative functional profiles of microbial communities on drifting microplastics and volcanic pumice
Source: ISME Commun. 2026 Jun 9;6(1):ycag158. doi: 10.1093/ismeco/ycag158 (PMC13374862; doi:10.1093/ismeco/ycag158)
Supplement: Supplementary_materials_ycag158 [file supplementary_materials_ycag158.zip › Supporting info_ISMECOMMUN-D-25-00630R3_07072026.docx]

Supporting information for

**Comparative Functional Profiles of Microbial Communities on Drifting Microplastics and Volcanic Pumice**

Shiye Zhao^1^*, Ryan P. Bos^2^, Ryota Nakajima^1^

^1^Institute for Earth and Materials Sciences, Japan Agency for Marine-Earth Science and Technology, Yokosuka, Kanagawa Prefecture, Japan

^2^Department of Organismic and Evolutionary Biology, Harvard University, Boston, MA, USA

***Corresponding author.** Institute for Earth and Materials Sciences, Japan Agency for Marine-Earth Science and Technology, 2-15 Natsushima-chō, Yokosuka, Kanagawa 237-0061, Japan.

E-mail: szhao@jamstec.go.jp

**Table S1. Information of the collected Plastisphere samples**

| Station | Sample name | Substrate type | Sample ID | Library kit used |
| --- | --- | --- | --- | --- |
| Station #1 | P-Sta1-2_S237 | Plastic | Plastic1 | MGIEasy FS |
| Station #1 | P-Sta1-4_S44 | Plastic | Plastic2 | MGIEasy FS |
| Station #1 | S-Sta1-6_S238 | Pumice | Pumice1 | MGIEasy FS |
| Station #1 | W1_S3 | Seawater | SW1 | MGIEasy FS |
| Station #2 | P-Sta2-10_S239 | Plastic | Plastic3 | MGIEasy FS |
| Station #2 | W2_S4 | Seawater | SW2 | MGIEasy FS |
| Station #5 | P1_S2 | Plastic | Plastic4 | MGIEasy FS |
| Station #5 | P2_S19 | Plastic | Plastic5 | MGIEasy FS |
| Station #5 | S1_S1 | Pumice | Pumice2 | MGIEasy FS |
| Station #5 | W4_S6 | Seawater | SW3 | MGIEasy FS |
| Station #7 | S2_S21 | Pumice | Pumice3 | Nextera XT |
| Station #7 | W5_S7 | Seawater | SW4 | MGIEasy FS |
| Station #7 | P3_S20 | Plastic | Plastic6 | MGIEasy FS |

**Table S2. PERMANOVA results for potential effects of DNA library kits used**

| Composition | Test | Results |
| --- | --- | --- |
| Taxonomy | ANOSIM | R = 0.311, *p* = 0.237 |
|  | PERMANOVA | R^2^ = 0.122, *p* = 0.154 |
| Function | ANOSIM | R = 0.253, *p* = 0.241 |
|  | PERMANOVA | R^2^ = 0.110, *p* = 0.245 |

**Table S3. Basic information obtained from the metagenomic sequencing analysis**

| Sample ID | Clean base (G) | No. of contig (>300 bp) | Predicted ORFs | Bacterial  proportion (%) | Eukaryote  Proportion (%) | Archaea  proportion (%) | *Staphylococcus* proportion (%) | *Bradyrhizobium* proportion (%) |
| --- | --- | --- | --- | --- | --- | --- | --- | --- |
| Plastic1 | 8.2 | 1592544 | 2015891 | 99.889 | 0.0013 | 0.098 | 0.00035 | 0.01 |
| Plastic2 | 11.4 | 1320374 | 1457213 | 99.637 | 0.002 | 0.361 | 0.17 | 0.004 |
| Plastic3 | 7.9 | 1437082 | 1351381 | 99.691 | 0.033 | 0.276 | 0.004 | 0.006 |
| Plastic4 | 12.4 | 2685691 | 3111829 | 99.556 | 0.014 | 0.430 | 0.002 | 0.009 |
| Plastic5 | 10.5 | 1955636 | 1804833 | 99.588 | 0.001 | 0.410 | 0.27 | 0.003 |
| Plastic6 | 7.9 | 1324159 | 1164692 | 99.587 | 0.002 | 0.411 | 0.27 | 0.002 |
| Pumice1 | 7.9 | 1639729 | 1874707 | 99.879 | 0.009 | 0.112 | 0.0002 | 0.009 |
| Pumice2 | 9.7 | 2214903 | 2645875 | 99.412 | 0.007 | 0.581 | 0.0009 | 0.01 |
| Pumice3 | 7.8 | 1026599 | 1104323 | 99.766 | 0.000 | 0.234 | 0.14 | 0.002 |
| SW1 | 10.0 | 2248843 | 2806808 | 99.909 | 0.000 | 0.091 | 0.0005 | 0.001 |
| SW2 | 9.2 | 1737455 | 2240912 | 96.468 | 0.000 | 3.531 | 0.002 | 0.001 |
| SW3 | 9.9 | 2355031 | 2845091 | 99.667 | 0.001 | 0.333 | 0.002 | 0.001 |
| SW4 | 11.7 | 2923544 | 3453465 | 99.474 | 0.002 | 0.524 | 0.003 | 0.002 |

**Table S4. Kruskal-Wallis test results of comparing the abundances of each obligate hydrocarbon-degrading bacteria genera**

| Genera | *chi-squared* | *P* |
| --- | --- | --- |
| *Alcanivorax* | 2.58 | 0.27 |
| *Erythrobacter* | 5.88 | 0.053 |
| *Flavobacterium* | 1.44 | 0.49 |
| *Oleiphilus* | 0.54 | 0.76 |
| *Ketobacter* | 1.64 | 0.44 |
| *Parvibaculum* | 0.35 | 0.84 |
| *Pseudomonas* | 5.36 | 0.07 |
| *Psychrobacter* | 3.54 | 0.17 |
| *Psychromonas* | 4.12 | 0.13 |


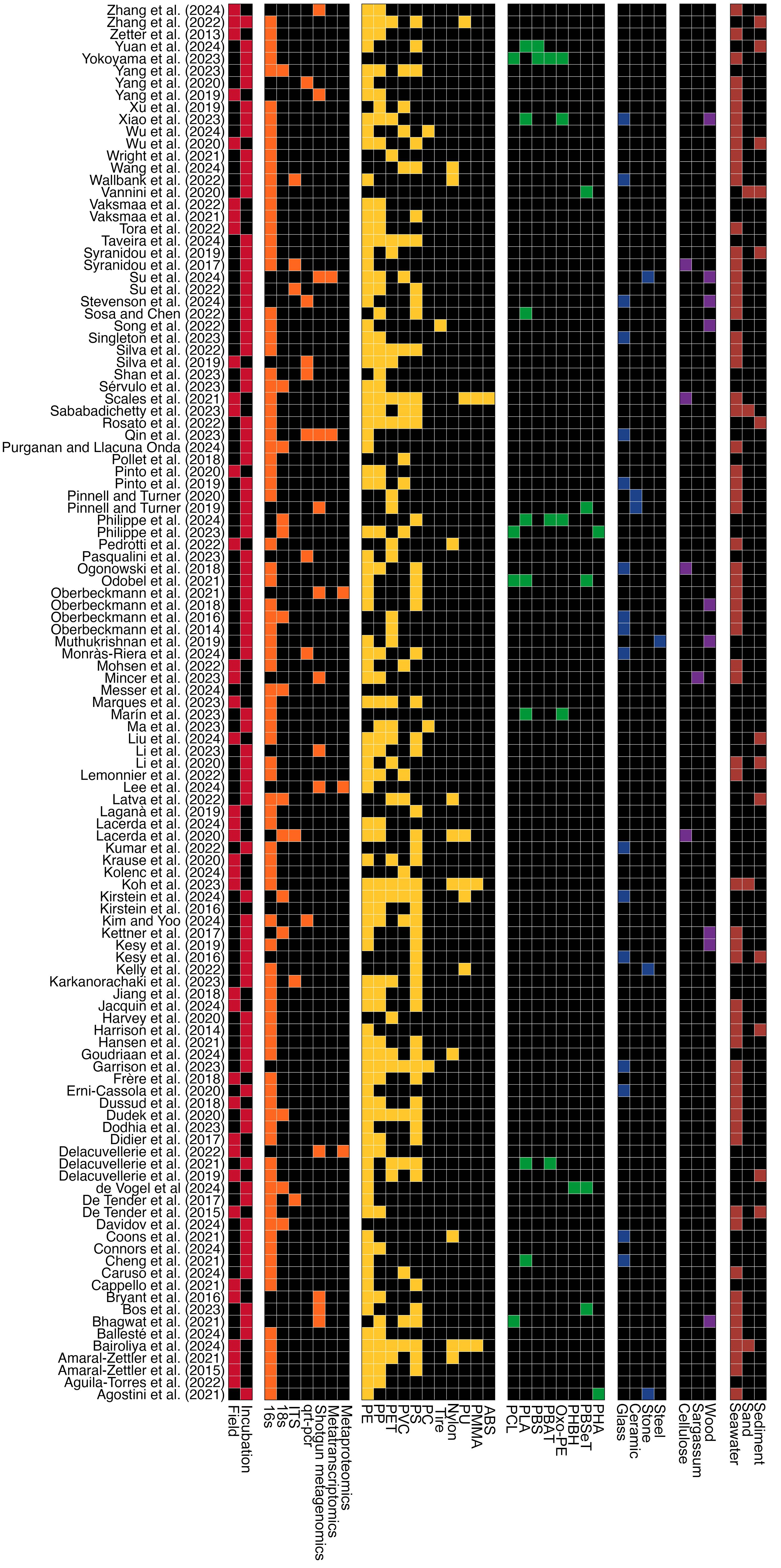


**Fig. S1** Study designs of 122 studies (Data spreadsheet 1) that analyzed the Plastisphere via sequencing techniques on marine plastic debris. Colored and black tiles indicate ‘Yes and No’. These factors include whether the samples were gotten through in-situ incubation or collection from the sea; whether the Plastisphere was analyzed via amplicon sequence (16S, 18S, or ITS rRNA) or shotgun metagenome sequencing, meta-transcriptomics and meta-proteomics; whether biofilms on conventional (PE: polyethylene, PP: Polypropylene, PET: polyester, PS: polystyrene, PVC: Polyvinyl chloride, PC: polycarbonate, PU: polyurethane, PMMA: polymethyl methacrylate, ABS: acrylonitrile-butadiene-styrene, Tire, and Nylon) or degradable plastic (PCL: polycaprolactone, PLA: polylactide acid, PBS: polybutylene succinate, PBAT: polybutylene adipate terephthalate, PHBH: poly3-hydroxybutyrate-co-3-hydroxyhexanoate, PBSeT: polybutylene sebacate-co-terephthalate, PHA: polyhydroxyalkanoates and Oxo-PE) polymer types were analyzed; whether the negative (glass beads, ceramic, stone and steel) and positive (cellulose, sargassum and wood), and background (seawater, sand and sediment) controls of Plastisphere were included. Further information including summaries of aims and key findings, materials tested, primers used, and sequencing accession numbers, is available in Data spread 1.


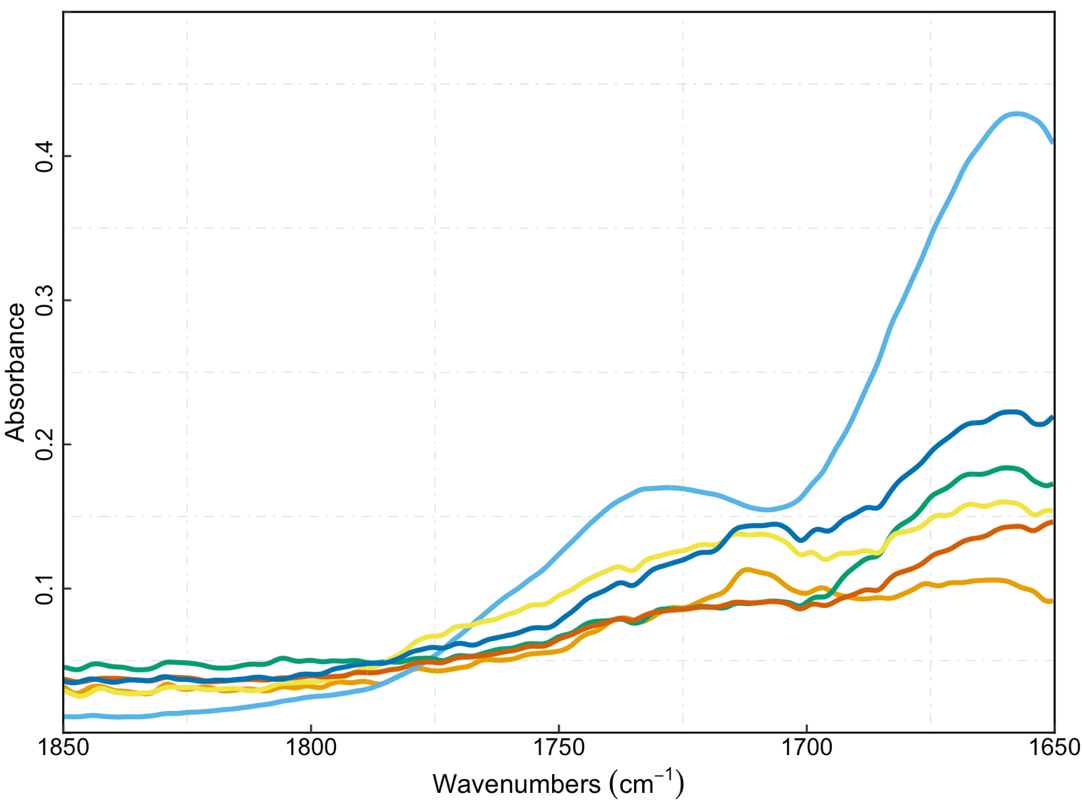


**Fig. S2** The absorbance of the carbonyl species of each microplastic particles in the range of 1850–1650 cm^-1^.


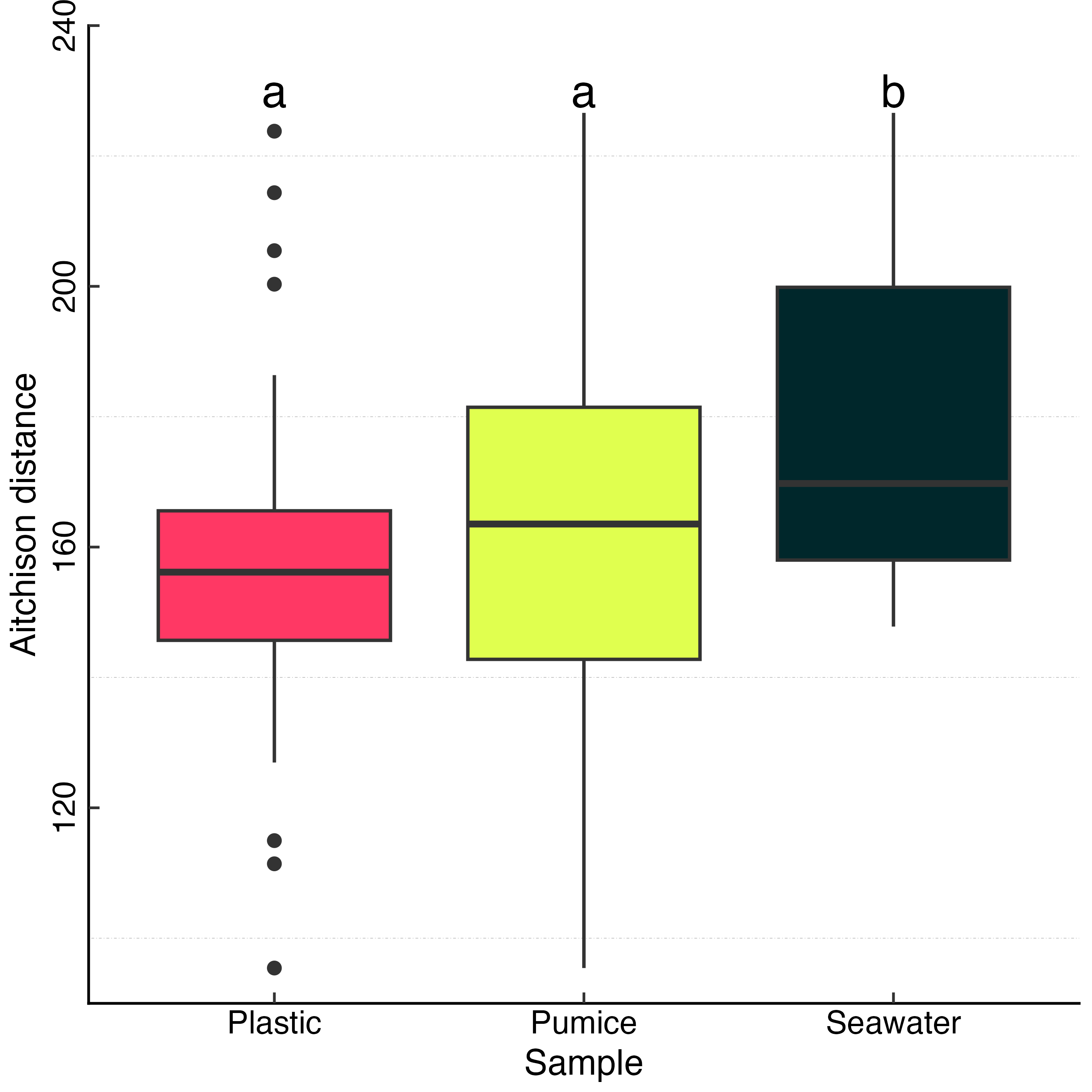


**Fig. S3** Aitchison distance reflects the similarity of bacterial communities between plastic, pumice and seawater sample. Significance of results (*p* = 0.005) was evaluated using Kruskal-Wallis and Mann-Whitney U tests and labeled using different letters. The center line represents the median; box limits indicate the first and third quartiles; and whiskers show 1.5 times the interquartile range.


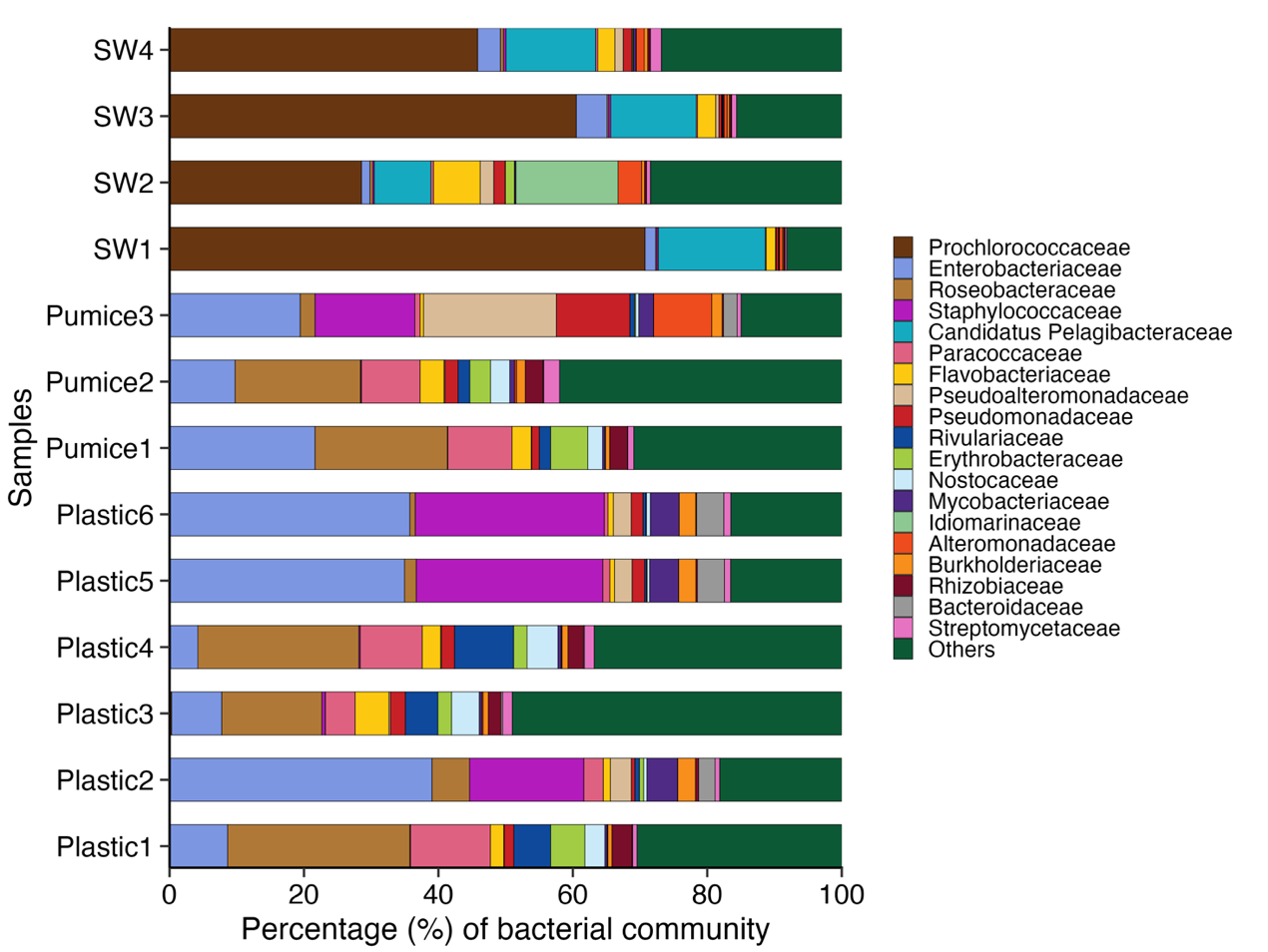


**Fig. S4**Bar chart showing the relative abundance of bacterial groups at the family-level. Families with abundances of <1% in one sample are shown in ‘Others’.

**
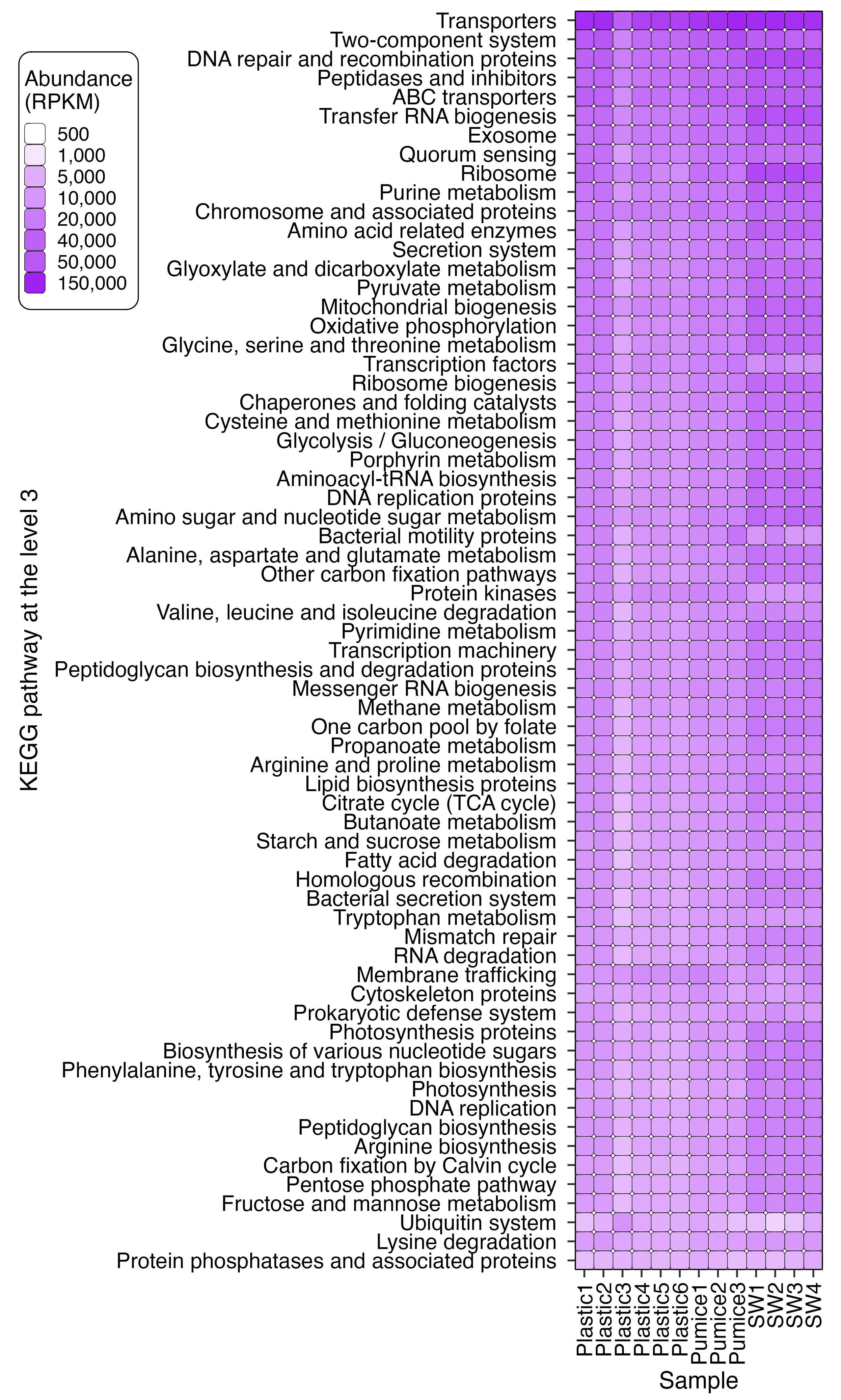
**

**Fig. S5** Profiles of 50 most abundant KEGG pathways from different substrates (plastics, pumices and seawater). The abundance of KEGG pathway at the category level 3 was represented as RPKM. The profiles were prepared by assembling the identified KOs into broader functional categories at different functional levels.

**
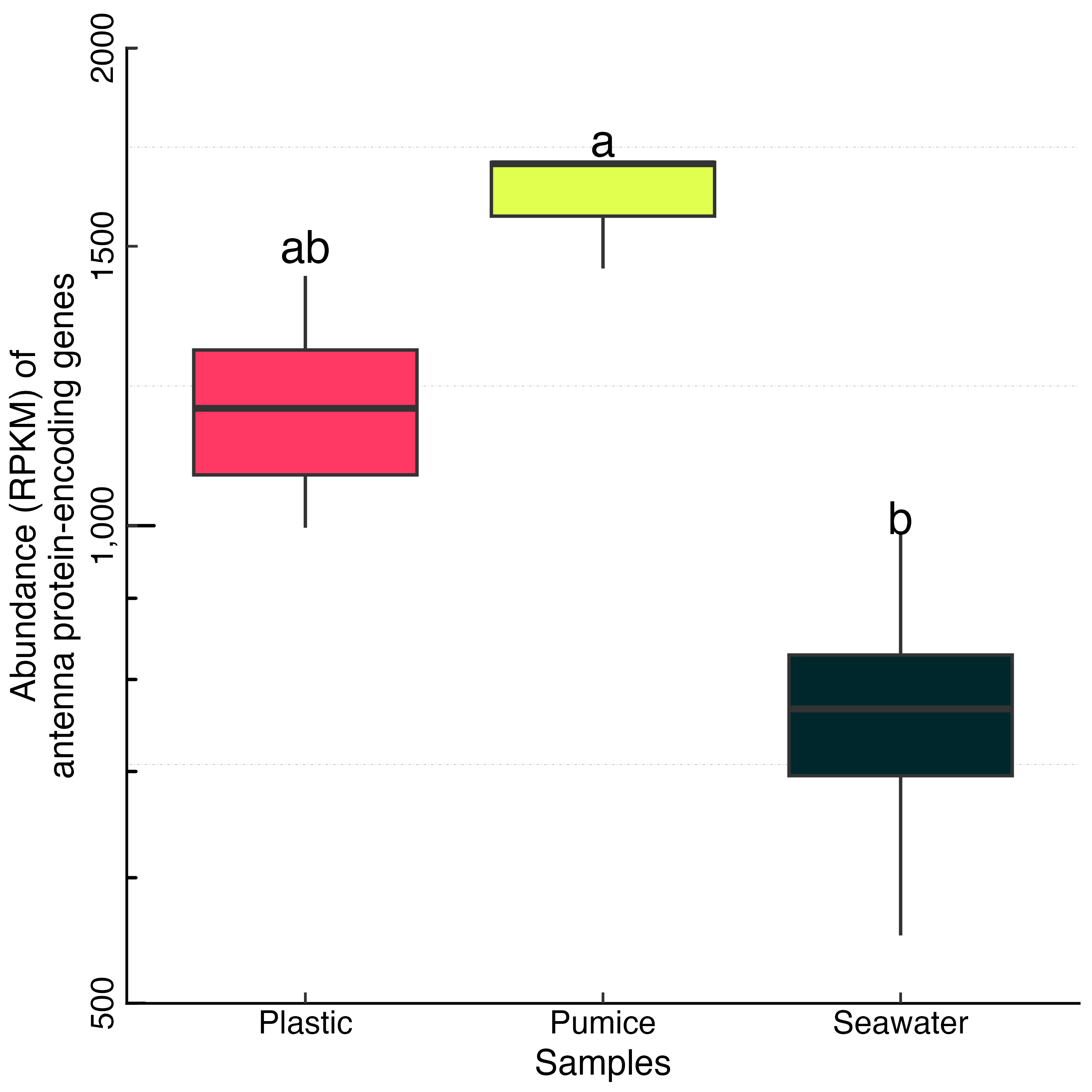
**

**Fig. S6** The gene abundance level of light-harvesting antenna proteins. Significance of results (chi-squared = 10.385, *p* = 0.005) was evaluated using Kruskal-Wallis and Mann-Whitney U tests and labeled using different letters. The center line represents the median; box limits indicate the first and third quartiles; and whiskers show 1.5 times the interquartile range. Outliers are indicated as black dots.

**
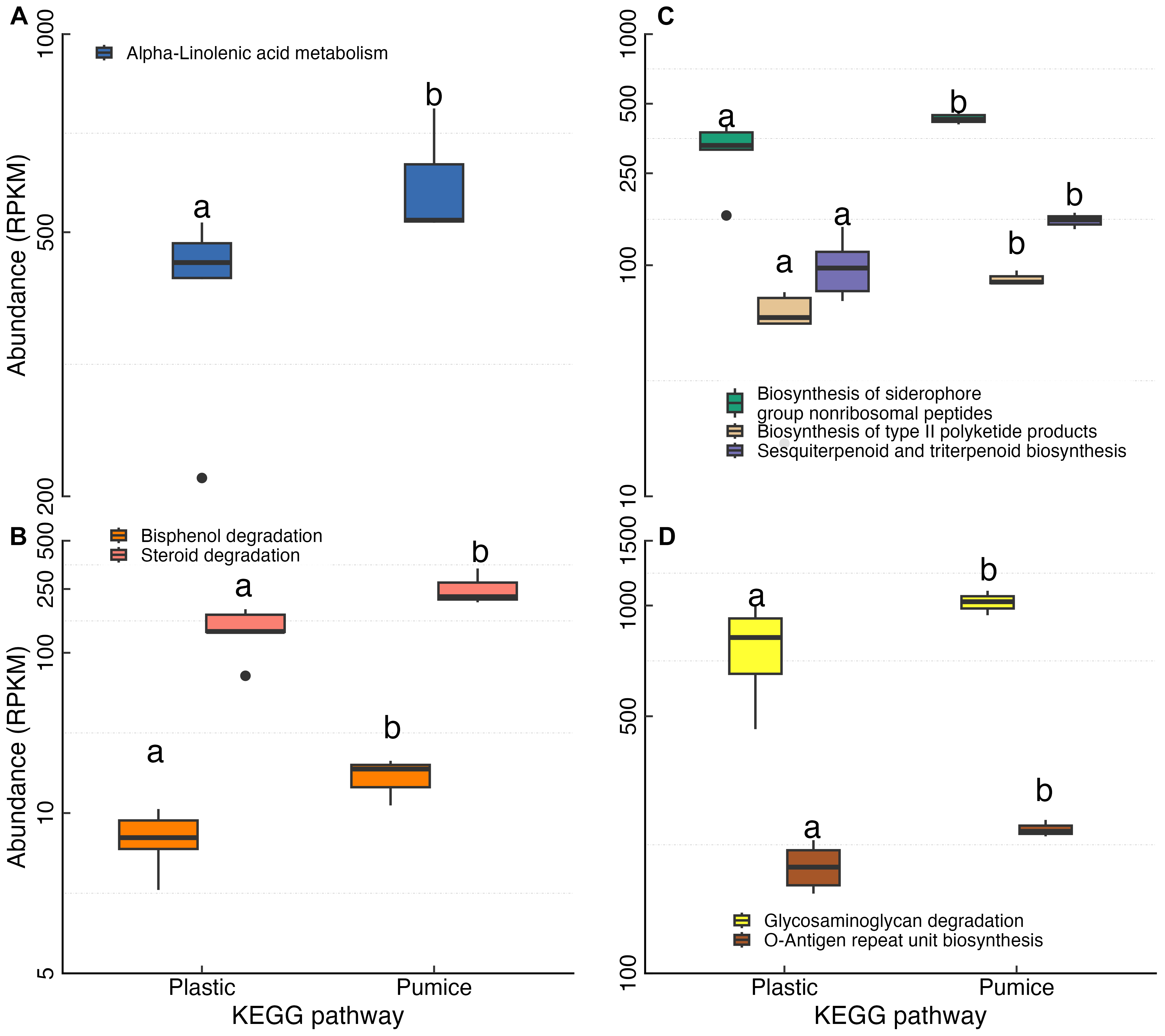
**

**Fig. S7** Comparison of the abundance (represented by RPKM values) of KEGG pathways at the category level 3 in the plastic- and pumice-attached metagenomes. A, B, C and D indicates the at the category level 2: Lipid metabolism, Xenobiotics biodegradation and metabolism, Metabolism of terpenoids and polyketides, Glycan biosynthesis and metabolism. The median values are marked with a bold vertical line, and whiskers represent the minimum and maximum values. Significance of results (p < 0.05) was evaluated using Mann-Whitney U tests .and labeled using different letters. The box limits indicate the first and third quartiles; and whiskers show 1.5 times the interquartile range. Outliers are indicated as black dots.

**
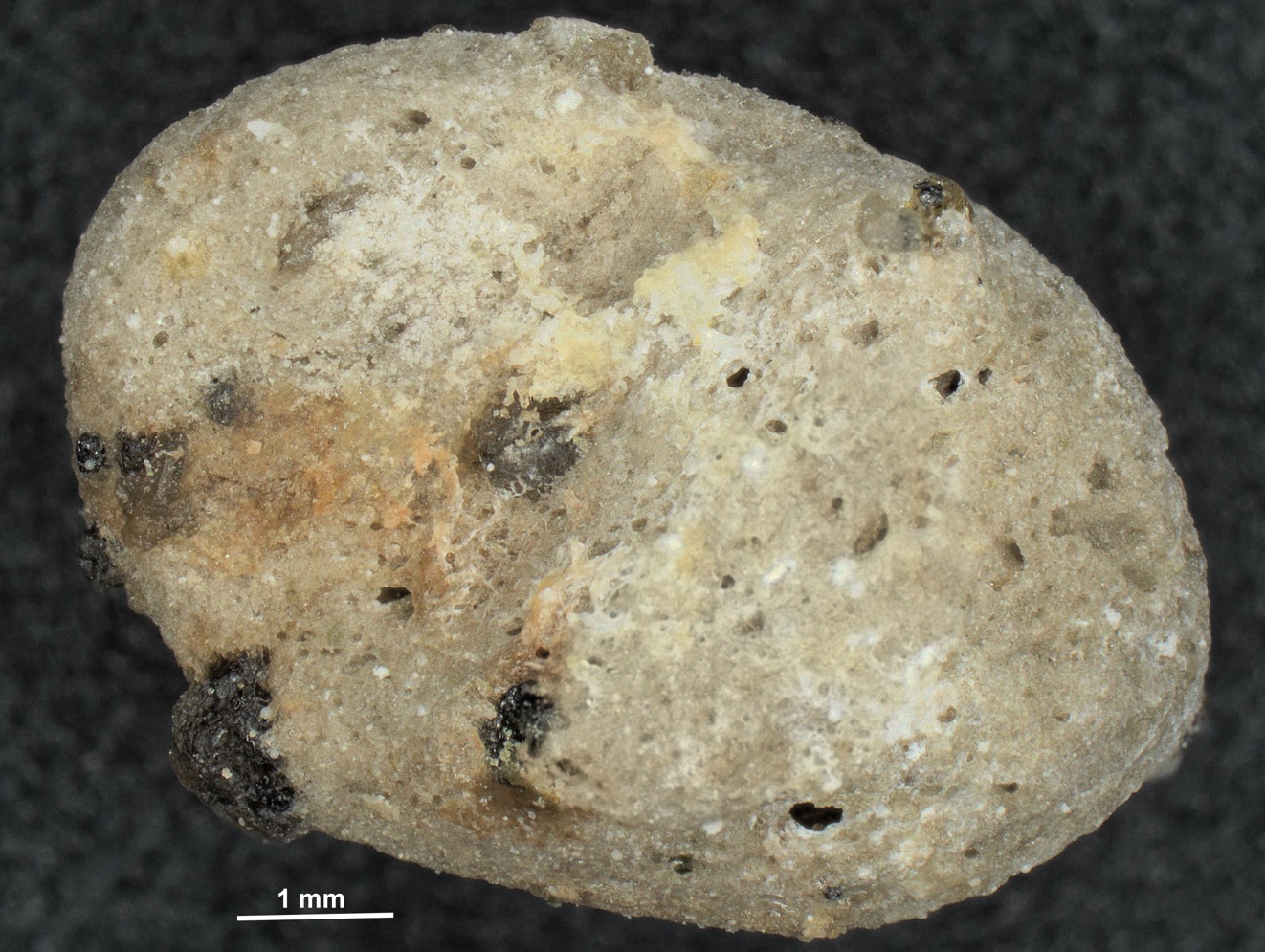
**

**Fig. S8** A micrograph of the pumice particle


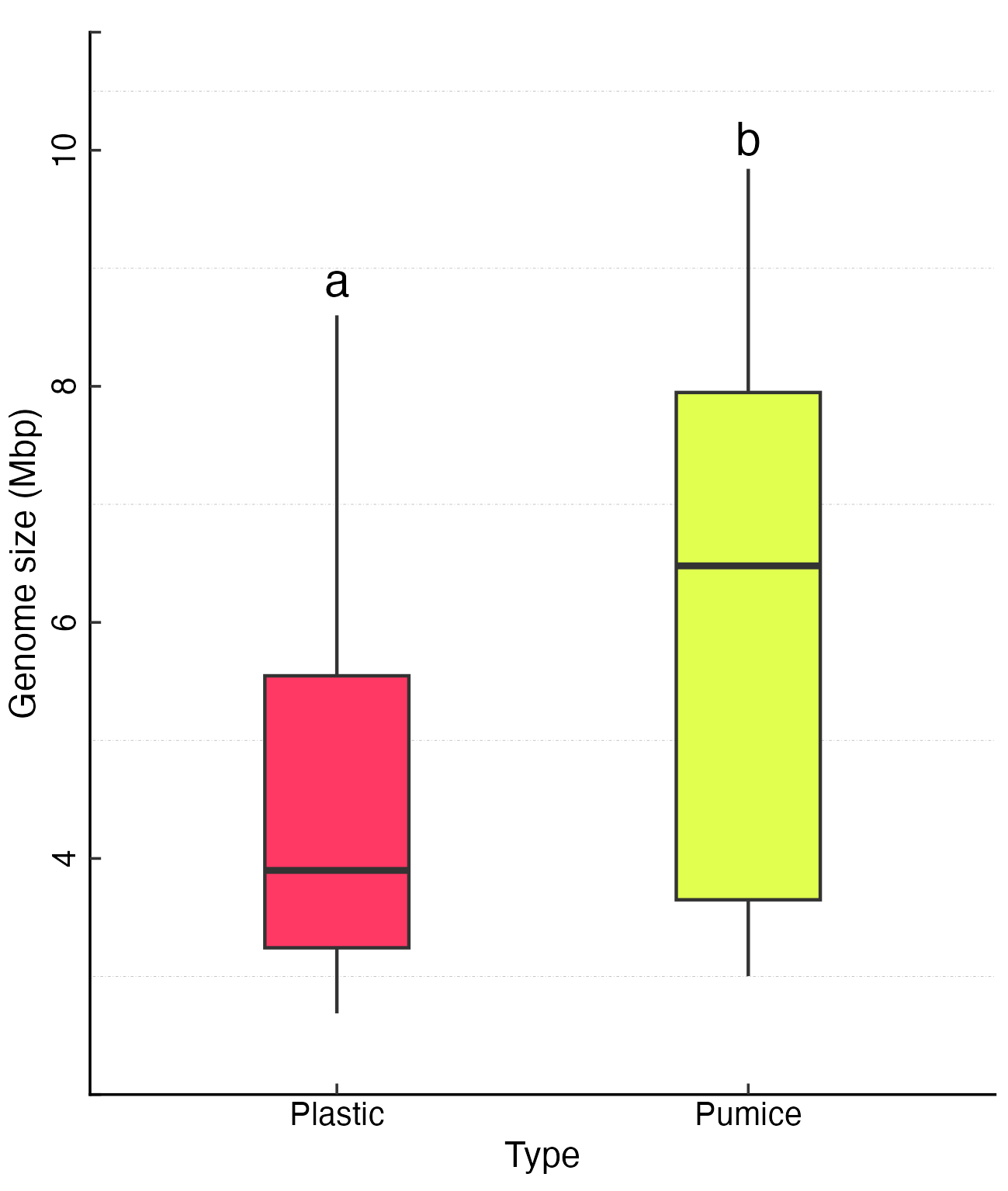


**Fig. S9** Variances of genome size in MAGs from plastic and pumice metagenome sample. The median values are marked with a bold vertical line, and whiskers represent the minimum and maximum values. Significance of results (*p* = 0.03 < 0.05) was evaluated using *Mann-Whitney U tests* and labeled using different letters. The center line indicates the median; the box limits indicate the first and third quartiles; and whiskers show 1.5 times the interquartile range.


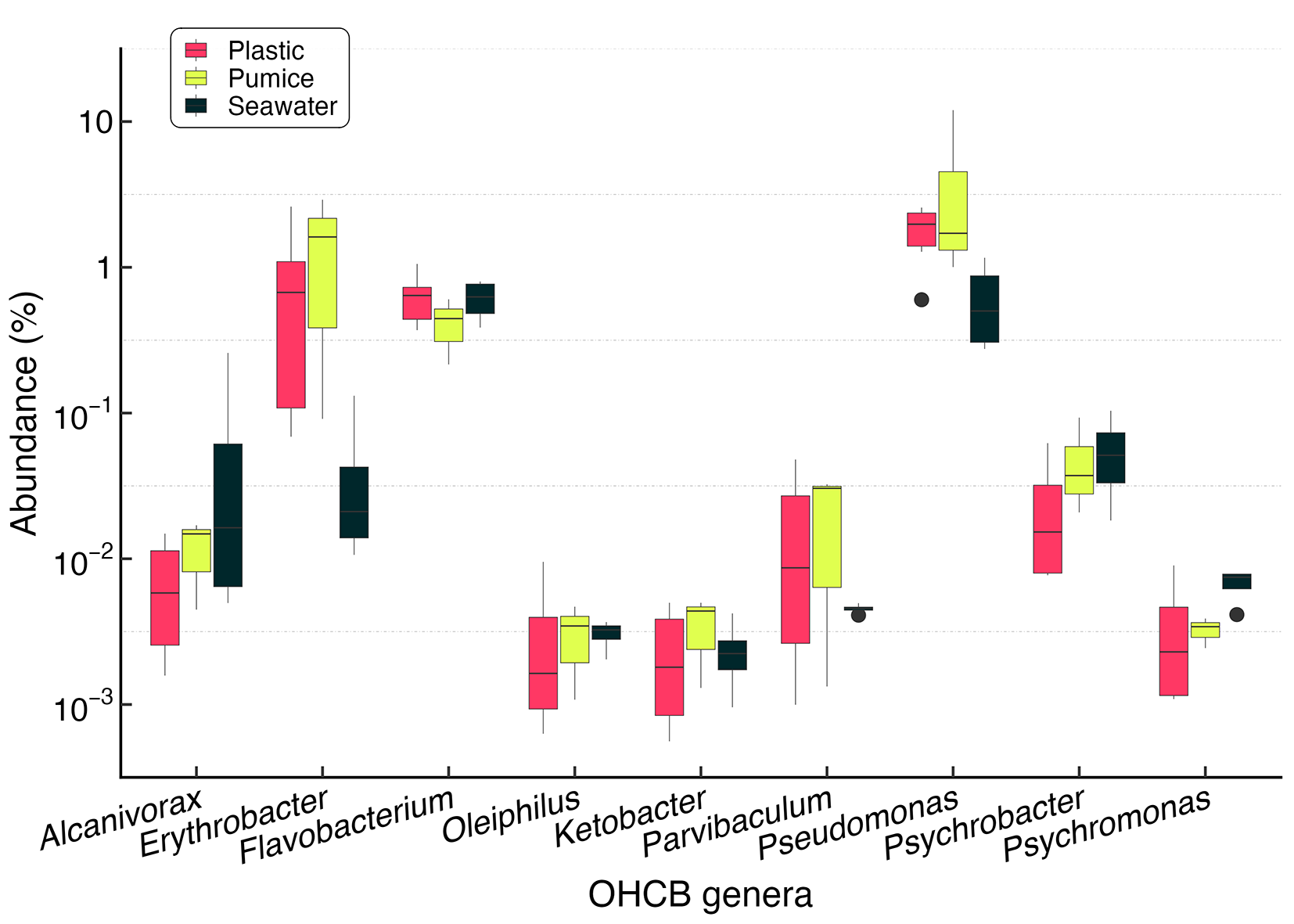


**Fig. S10** The relative abundance of the obligate hydrocarbon-degrading bacteria identified on plastics, pumices and in the seawater. The center line indicates the median; box limits represent the first and third quartiles; and whiskers extend to 1.5 times the interquartile range. Statistical comparisons were performed using *Kruskal-Wallis test* (see Table S4 for details).


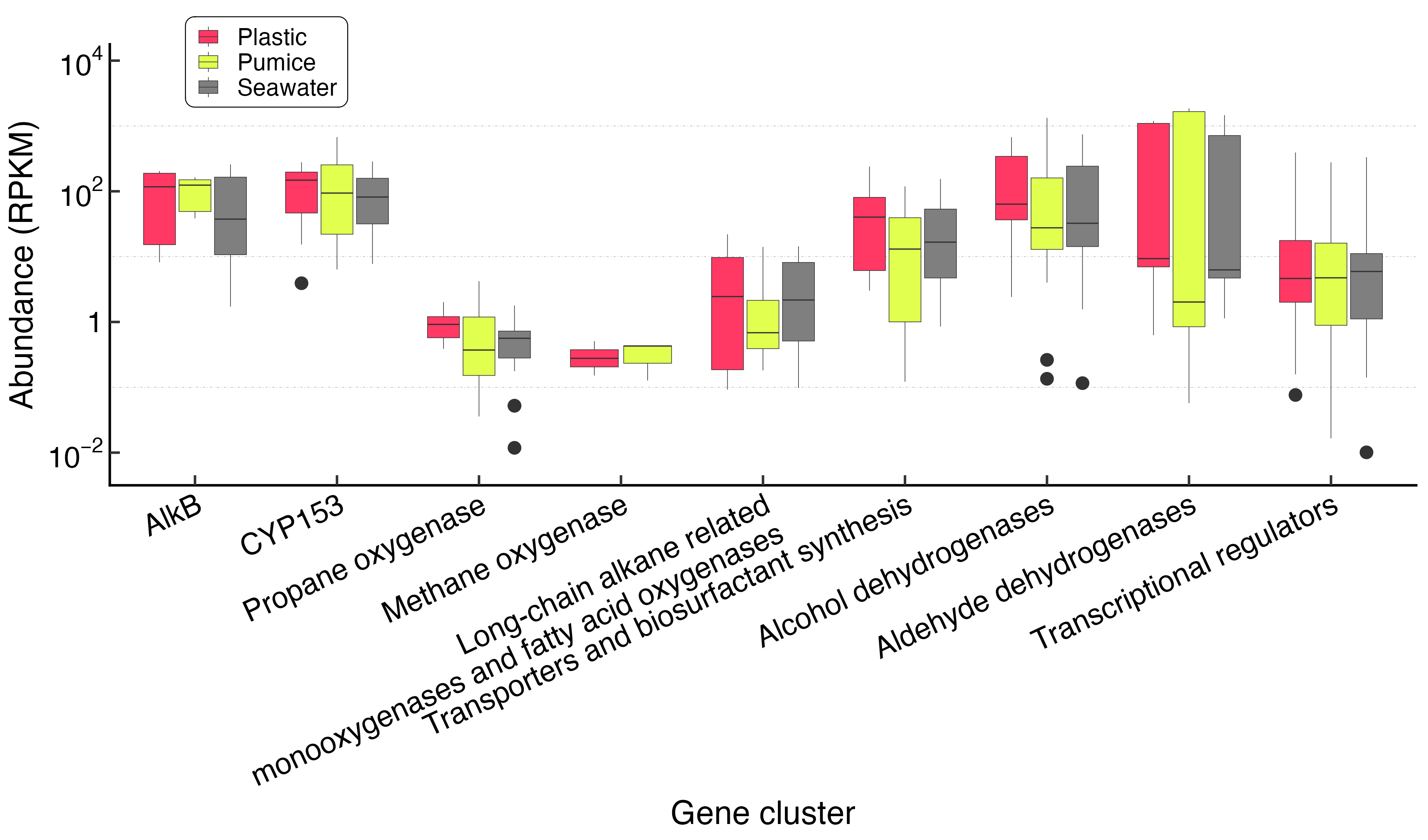


**Fig. S11** The relative abundance of nine selected gene groups associated alkane and fatty acid degradation on plastics, pumices and in the seawater. The center line represents the median; box limits indicate the first and third quartiles; and whiskers show 1.5 times the interquartile range.


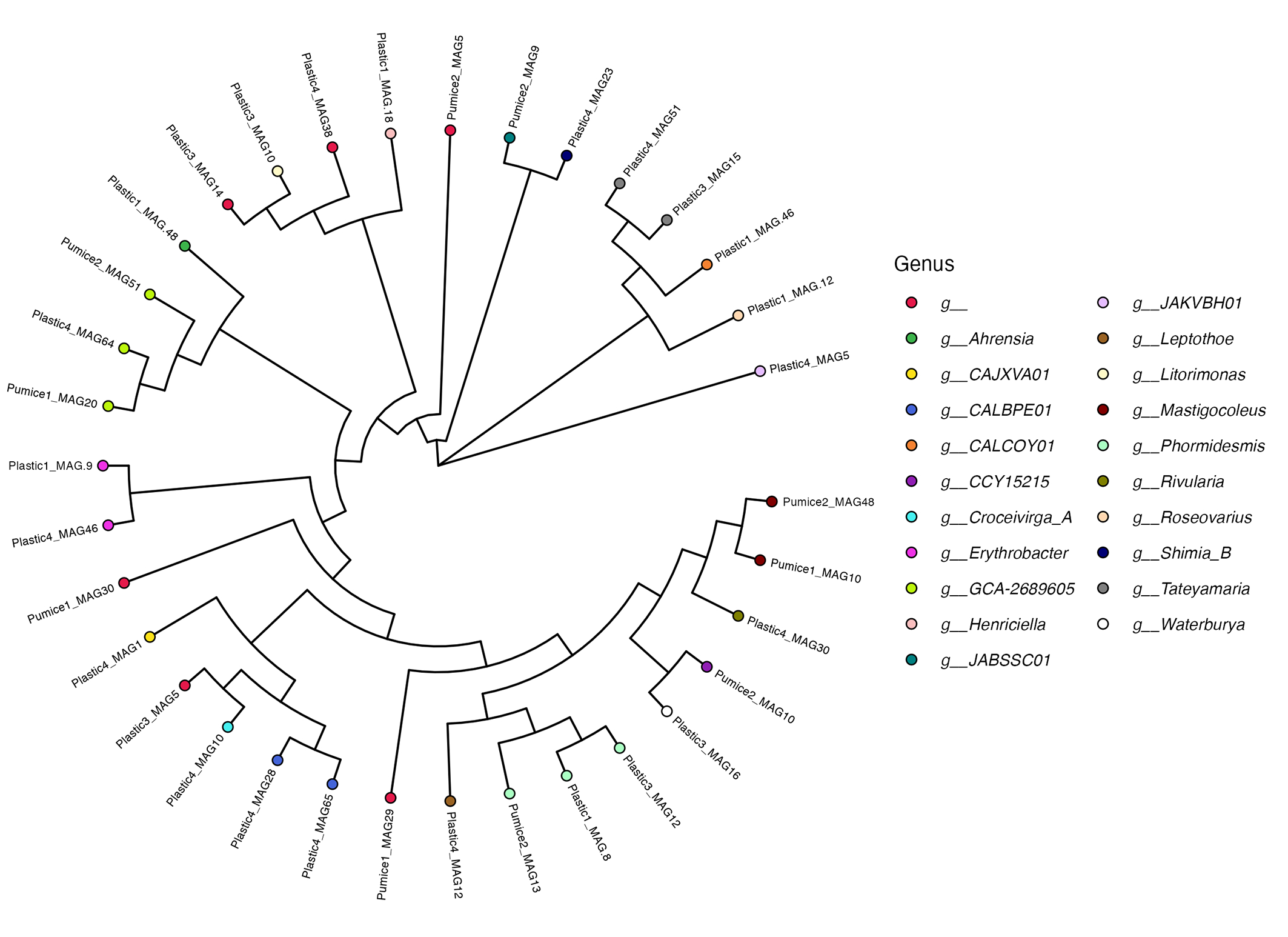


**Fig. S12** Phylogenetic tree of the 34 MAGs inferred from a concatenated alignment of 120 bacterial marker genes. Tip labels indicate the sample origin and corresponding bin number of each MAG.


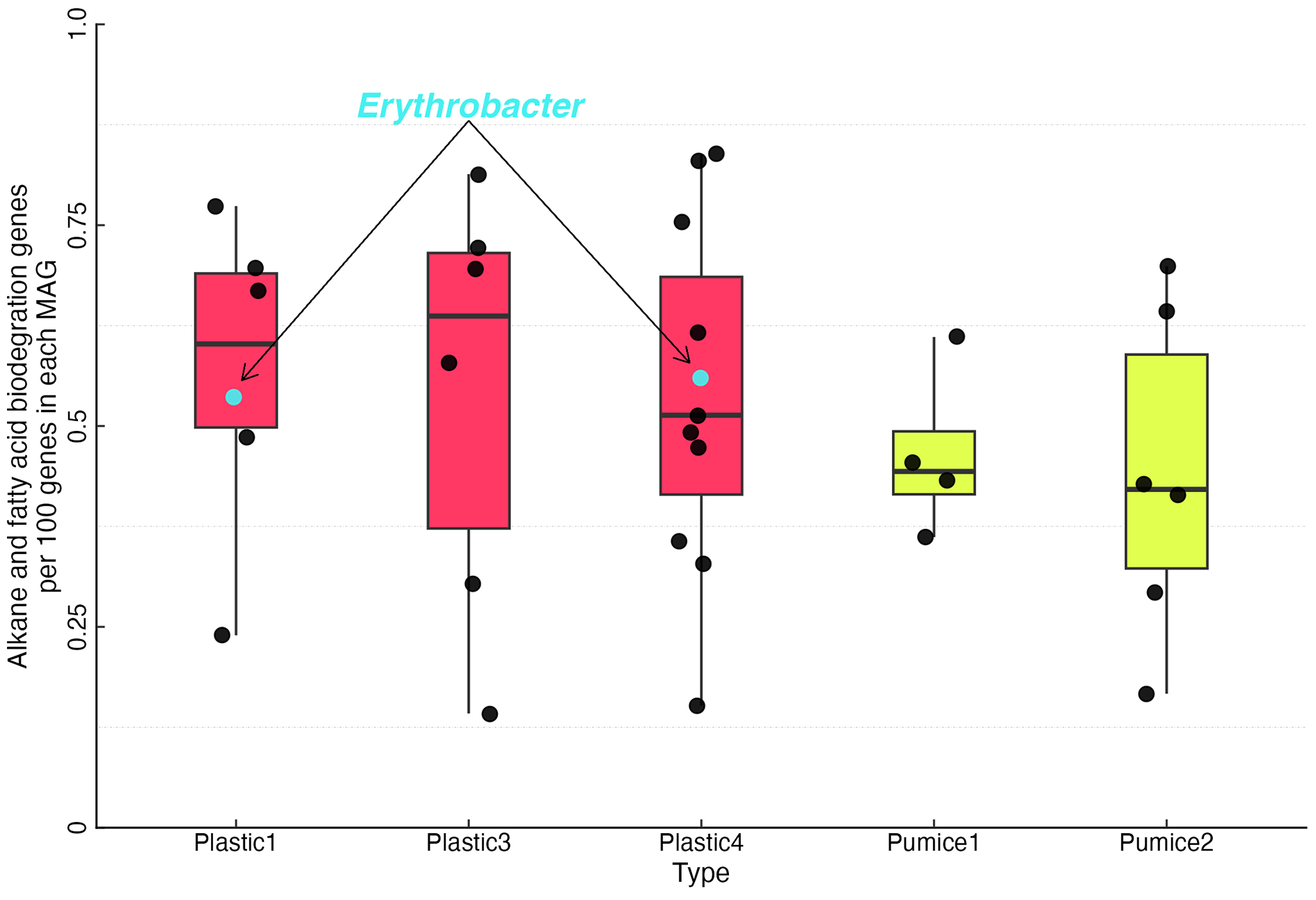


**Fig. S13** Relative abundances of selected genes involved in alkane and/or fatty acid degradation across the 34 MAGs in their corresponding samples. Light blue dots represent MAGs assigned to the genus *Erythrobacter*, an obligate hydrocarbon degrader. No significant differences in gene abundances among samples were detected (Kruskal-Wallis test, chi-squared = 2.08, *p* = 0.72). The center line indicates the median; box limits indicate the first and third quartiles; whiskers extend to 1.5 times the interquartile range.


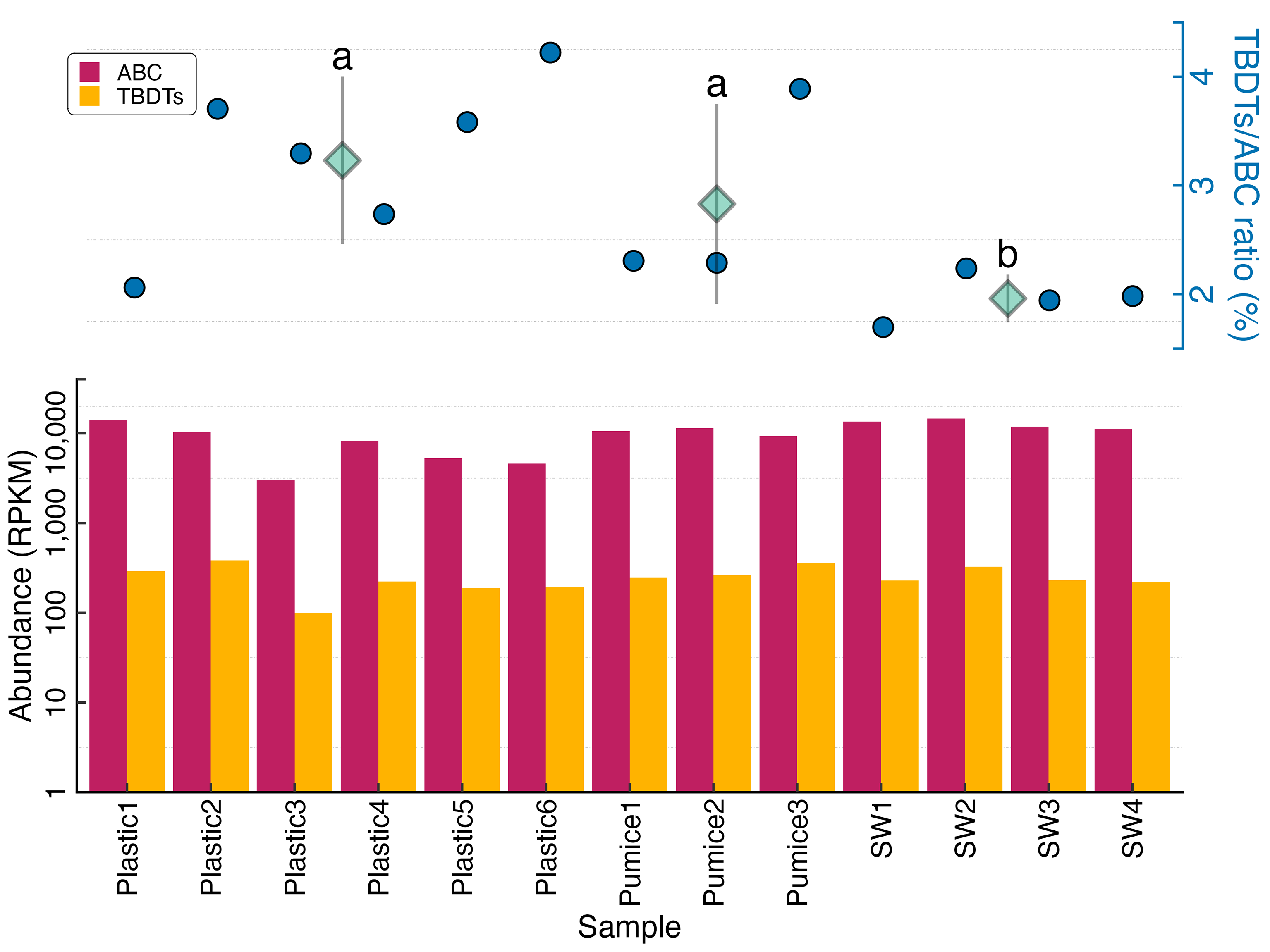


**Fig. S14** Prokaryotic expression of ATP-binding cassette (ABC) transporters and TonB-dependent transporters (TBDTs) in plastic, pumice, and seawater (SW) samples. The lower panel displays a bar plot of the relative gene abundances (RPKM) of both transporters in each sample. The upper panel presents a point plot of TBDT/ABC ratios per sample and the mean ratio with standard deviation for each sample type. Different letters above the mean ratios indicate significant differences between sample types, as determined by a Kruskal-Wallis test (*p* = 0.031; *chi-squared* = 6.97).


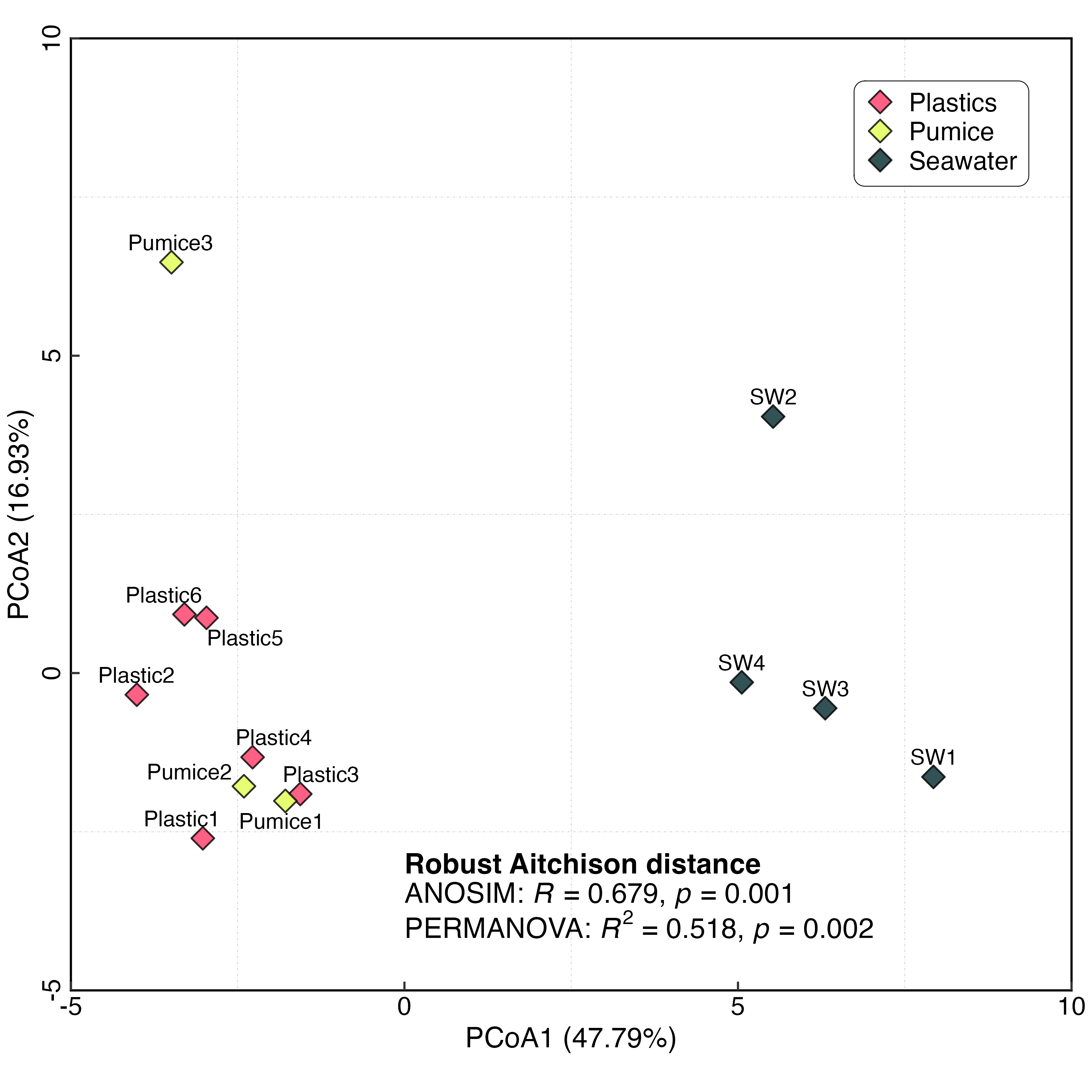


**Fig. S15** GH families genes (RPKM) abundances-based PCoA plots of the Robust Aitchison’s distance. Results of ANOSIM and PERMANOVA tests are shown in the box.
