# Supplementary figures and images for: Comparative functional profiles of microbial communities on drifting microplastics and volcanic pumice
Source: ISME Commun. 2026 Jun 9;6(1):ycag158. doi: 10.1093/ismeco/ycag158 (PMC13374862; doi:10.1093/ismeco/ycag158)

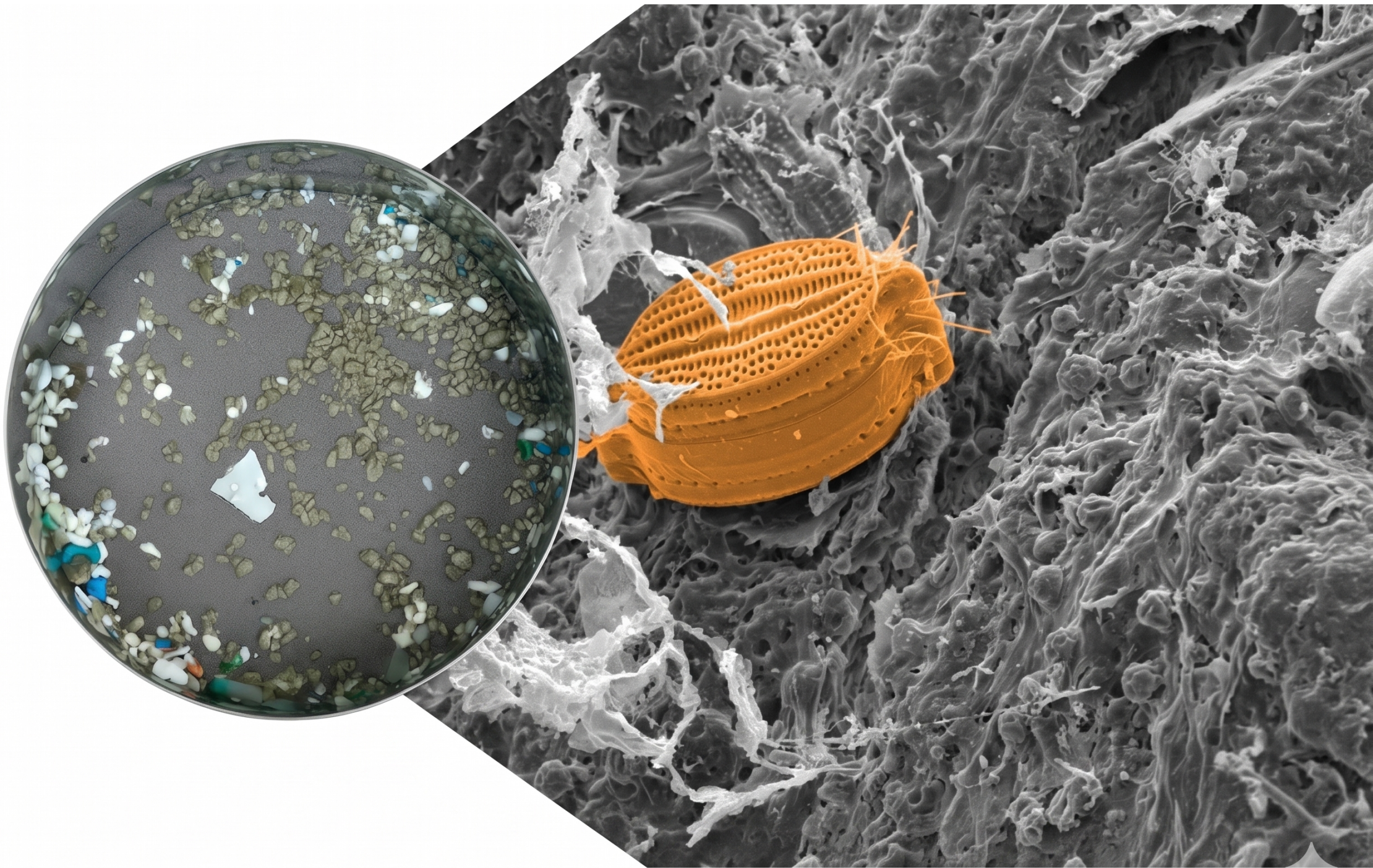

Supplement: Supplementary_materials_ycag158 [file supplementary_materials_ycag158.zip › Microbes on Plastics & Pumices.pdf]
